# Osmolyte Structural and Thermodynamic Effects Across the Protein Folding Landscape

**DOI:** 10.1021/jacsau.5c00813

**Published:** 2025-10-13

**Authors:** Ander Francisco Pereira, Leandro Martínez

**Affiliations:** Institute of Chemistry and Center for Computing in Engineering & Science, 344102 Universidade Estadual de Campinas (UNICAMP), 13083-861 Campinas, São Paulo, Brazil

**Keywords:** protein folding, osmolyte, transfer free energy, Kirkwood–Buff theory, minimum-distance distribution function

## Abstract

In this work, the structure and thermodynamics of urea and trimethylamine N-oxide (TMAO) preferential interactions across the complete folding landscape of the β-sheet SRC Homology 3 (SH3) domain and the helical B domain of protein A (BdpA) are characterized. There is a high correlation between preferential interactions and the surface area of the denatured states, despite the chemical nature of the exposed surfaces upon denaturation differing from those of native states. For SH3, the denaturation always proceeds with an increase in surface area, such that the qualitative effect of cosolvents on the stability of all denatured states can be inferred from the preferential interactions in the native state. On the other hand, for BdpA, partially denatured states exist that are destabilized by urea; thus, unfolding pathways can be modulated by the cosolvent. Both urea and TMAO form hydrogen bonds with the proteins, which are on average weakened upon denaturation, and nonspecific interactions, which are strengthened in unfolded structures. Backbone, side chain, and specific residue contributions to distribution functions are obtained, illustrating, for instance, the crucial participation of urea-backbone and nonpolar–cosolvent interactions in the solvation mechanisms, on a residue basis. Obtaining these results was possible using a novel computational pipeline to represent solvation structures throughout complete folding landscapes by means of coarse-grained and atomistic simulations and, crucially, the analysis of solvation using minimum-distance distribution functions and the Kirkwood–Buff solvation theory. Cosolvent effects on transfer free energies match experimental data within 1 kcal mol^–1^, supporting the nuanced description of the osmolyte-protein interplay. The proposed methods can be applied to the study of solvation structure and thermodynamics in other complex molecular systems undergoing large conformational variations, such as nonbiological macromolecules and aggregates.

## Graphical abstract

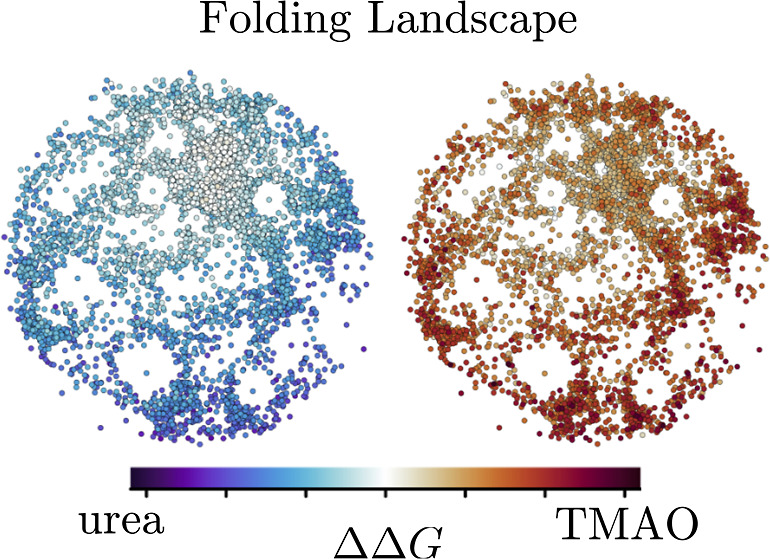

## Introduction

Urea and trimethylamine N-oxide (TMAO) are osmolytes that exert different effects on protein stability, with urea typically acting as a denaturant and TMAO as a protectant.^1–6^ These effects can be analyzed, at a first level of approximation, from interactions with the protein in its native state: osmolyte exclusion from the protein surface favors compact structures, typically linked to folded forms.^7,8^ Conversely, preferential protein-osmolyte interactions favor the exposure of the protein residues and thus denaturation.^9,10^ These are, in fact, the well-known behaviors of urea and TMAO, providing the foundation for the understanding of their effects on protein stability.^6,10,11^ This rationale is oversimplified for proteins because denaturation alters the overall chemical nature of the protein surface. Therefore, interactions observed in the native state do not fully capture the complex interplay between water and cosolvents in the unfolded ensemble.^6,12–14^

Obtaining preferential interaction parameters for multiple protein conformations allows for quantitative analysis of the impact of cosolvents on folding equilibrium.^10,12,15^ Yet, to obtain the thermodynamic cosolvent effect on the folding equilibria requires either directly measuring equilibrium constants in both pure solvent and cosolvent solutions or determining the transfer free energies of the native and denatured states.^2^ The experimental characterization of the folding equilibrium is challenging because the conformations of native and unfolded ensembles may differ in different protective and denaturant conditions, and experiments depend on a reduced variable to classify the ensembles: the protein activity, the secondary structure content, a specific NMR signal, etc. From a computational modeling perspective, the challenges are (1) the proper representation of the interactions; (2) the difficulty in sampling diverse macromolecular conformational states; and (3) the molecular representation of the solvation in structural diverse ensembles and computing observables. In the context of the present work, the force fields developed for reproducing protein preferential interactions of urea^16^ and, more recently, for TMAO,^17^ address the first challenge, and we build our work on top of these developments.

The challenge of representing diverse macromolecular conformations has been tackled with roughly two approaches: simulating small models of peptides or amino acid residues,^17–20^ or through the generation of unfolded states of larger proteins by means of long simulations in denaturing conditions^14^ or with constructs based on experimental data.^13^

The simulations with small protein models allowed the quantitative computation of observables such as preferential interactions and transfer free energies, and provided many essential insights into the mechanisms of urea and TMAO action on protein stability. In a key study, Pettitt and co-workers obtained a detailed characterization of the solvation of a helical decalanine peptide.^18^ They focus on the competing roles of TMAO and urea and were able to show that denatured states of the helix display enhanced preferential solvation by urea. TMAO was also observed to dehydrate the helix, although to a lesser extent, such that the transfer free energies follow the expected trends. They obtain transfer free energies by explicitly computing, using generalized replica exchange simulations, solvation free energies at varying cosolvent concentrations. More recently, Shea and co-workers revisited the urea-TMAO competing mechanisms on protein stability by modeling peptides in solutions of urea and TMAO.^17^ Notably, they reparametrized the TMAO force-field, obtaining preferential exclusion consistently with experimental data. In parallel, simulations of small peptides in urea showed that urea is preferred over water by all but charged amino acid side chains, supporting that direct hydrophobic interactions are an essential part of the urea-induced denaturation thermodynamics.^21^

To demonstrate that the conclusions obtained on small peptide constructs can be transferred to proteins, simulations of protein denaturation were necessary. The key difference is that, for larger proteins, the chemical nature of the residues exposed upon denaturation differs, on average, from that of the surface of the native state. Effectively, since urea interacts more favorably than water with most side chains,^21^ the exposure of the core of the protein leads to urea early intrusion into the protein globule and progressively enhances van der Waals interactions, as demonstrated by Berne and co-workers.^14^ These observations are confirmed by the simulation of experimentally supported denatured states of Ubiquitin in concentrated urea solutions.^13^

The missing link between the small-model and large-protein simulations is the ability to comprehensively describe the solvation structure and thermodynamics of denatured states. The computation of protein-cosolvent preferential interaction parameters by means of Kirkwood–Buff theory is a well-developed field.^22,23^ Nevertheless, obtaining both a molecular picture of solvation and the solvation thermodynamic parameters for denatured states remains a challenge. A step forward was the demonstration by Martínez and Shimizu that the connection between the solvation shell picture of solute–solvent interactions and the solvation thermodynamics can be obtained for structures of arbitrary shape using minimum-distance distribution functions (MDDFs).^24^ We also developed specialized computational analysis tools to leverage this concept,^25^ allowing the study of structurally complex molecular environments with thermodynamic rigor.^12,26^

Given that MDDFs can be used to characterize the solvation of denatured states, we here exploit their combination with structure-based models (SBMs)^27^ and atomistic simulations to generate complete folding landscapes of proteins in solvent mixtures. We characterize the solvation structures of two proteins with distinct topologies: the β-sheet SRC Homology 3 (SH3) domain^28^ and the α-helical B domain of protein A (BdpA)^29^ in aqueous urea and TMAO, and their preferential interaction coefficients and transfer free energies at the level of individual denatured states.

The contributions of the present study to the current understanding and modeling of osmolyte-protein interactions are 3-fold: first, from a mechanistic perspective, we show in unprecedented detail how the molecular interactions vary across the native and denatured protein states, understanding the nuanced competition between water and cosolvent for specific sites and chemical groups of the macromolecules. From a thermodynamic perspective, it is the first, to our knowledge, computation of transfer free energies of atomistic descriptions of denatured ensembles, with implications for the role of cosolvents on protein folding pathways. Third, the computational pipeline herein described can be applied to other complex macromolecular-solvent systems, particularly for leveraging the use of shape-adaptive distribution functions to connect the structure and thermodynamics of solvation.

## Results

### The Folding Landscape of Model Proteins

Understanding how cosolvents affect the protein folding free-energy surface requires the characterization of the protein conformational variability. The energy landscape (EL) theory^30,31^ provides a framework for modeling protein-folding mechanisms, and forms the basis for simulations using structure-based models (SBMs).^27^ SBMs are demonstrated to provide coarse-grained representations of the protein folding energy surfaces and structural ensembles of model proteins.^32–41^ In this work, SBMs are initially used to sample the conformational landscape of SH3 (a β-fold model, Figure 1A) and BdpA (a helical domain, Figure 1B).^42^ The folding mechanisms of these models have been extensively characterized by experimental and computational methods, including by SBM simulations.^32–36,42,43^

**Figure 1:**
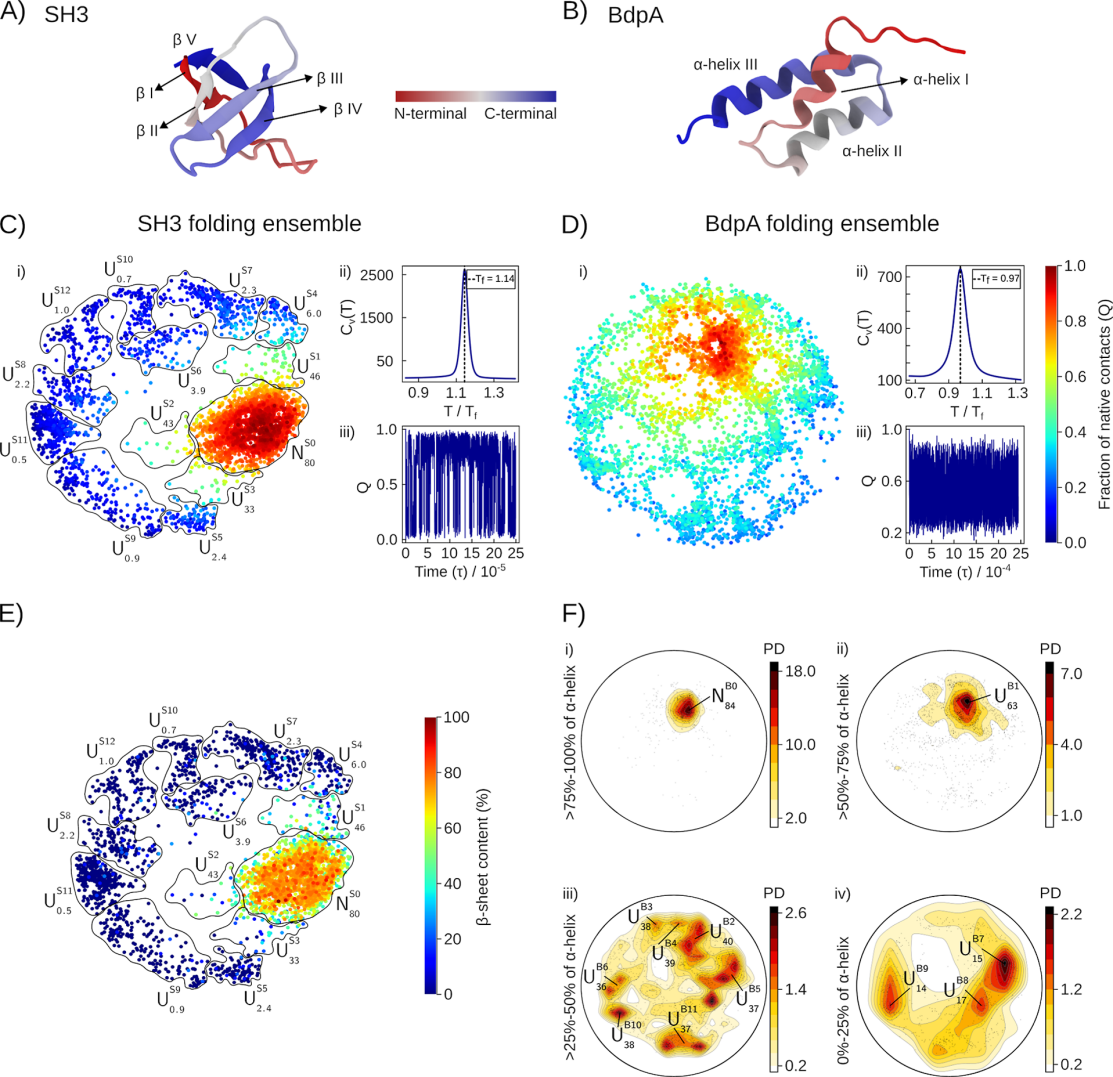
Folding ensembles of SH3 and BpdA. (A,B) Cartoon representation of SH3 ^28^ and BdpA^29^ native folds. (C,D) SBM folding ensembles characterized by (i) the ELViM projection, (ii) the specific heat (*C*_v_) as a function of temperature, and (iii) the fraction of native contacts (*Q*) as a function of time, in reduced units (τ). Each dot in (i) represents one structure in a color scale of *Q*, ranging from 0 (unfolded state) to 1 (folded state). (E) Folding projection of SH3 colored by secondary structure content, and definition of the conformational subsets. There is a high correlation between *Q* (panel C) and the secondary structure content. (F) Secondary structure dimension of the 2D projection for BpdA, which is required for classifying unfolded states.^42^ In Figures (E,F), labels are assigned to clusters of native (*N*) and unfolded (*U*) states, with a subscript indicating the average secondary structure content and the superscript the order of decreasing fraction of native contacts.

SBM simulations can be performed at the folding temperature of the model, obtaining multiple folding and unfolding events that allow the reconstruction of the folding free-energy surface. For SH3, the overall properties of the SBM sampling are shown in Figure 1C, and the protocol and characterization of the SBM folding are available in Supporting Information Figures S1 and S2. For BdpA, the characterization of the folding landscapes is shown in Figure 1D, and further details were recently published.^42^ Single sharp peaks in the *C*_v_(*T*) profiles (Figure 1C,D) indicate two-state folding mechanisms, but the folding transition is sharper for SH3 than for BdpA. In Figure 1C,D, panels (iii), the exhaustive sampling of native contacts (*Q*) over time can be observed, an indicator of the proper sampling of the conformational landscapes.

Visualization of the folding landscape requires techniques for dimensionality reduction. Thus, for each SBM simulation, the sampled conformations were projected in a two-dimensional space using the Energy-Landscape Visualization Method (ELViM).^35,44^ In panels (i) of Figure 1C,D, each dot represents a conformation obtained in the SBM simulation, and the distances of the dots in the projection are optimized to correlate with the dissimilarity of the models.^35^ The colors of the dots, from blue to red, represent completely unfolded (*Q* = 0) to completely folded (*Q* = 1) conformations. In Figure 1C,F labels are assigned to clusters of conformations: *N* and *U* represent the native basin and unfolded subsets, with a subscript indicating the average secondary structure content of the cluster, and the superscripts *S* or *B* for SH3 or BdpA followed by the classification of the cluster in terms of decreasing *Q*. For example, *N*_80_^S0^ is the native cluster of SH3, which displays on average 80% of the secondary structure of the crystallographic model. Alternatively, *U*_37_^B11^ is, in Figure 1F, an unfolded cluster of BdpA with 37% of its native secondary structure content, and the 11th cluster in decreasing amount of native contacts. This labeling will be used along the manuscript to reference the subsets of the folding landscape of the models. Comprehensive structural characterizations of each ensemble are shown in Supplementary Figures S3–S5, and Table S1.

Figure 1C,D show that the folded ensembles (in red) are clustered, as expected from the small native-state conformational variability. On the other hand, unfolded states are spread over the projection, because of their structural diversity. The folding variability of SH3 is captured by the 2D projection: there is a clear native-state basin (*N*_80_^S0^), separated from a range of unfolded states (*U*_6.0_^S4^ – *U*_1.0_^S12^) by only three intermediate sets of conformations (*U*_46_^S1^, *U*_43_^S2^, and *U*_33_^S3^). Furthermore, there is a high correlation between the fraction of native contacts (Figure 1C) and the secondary structure content (Figure 1E).

In contrast, the folding space of BdpA displays multiple pathways connecting the native (*N*) and unfolded (*U*) states via structures with similar *Q* (Figure 1D). Furthermore, partially unfolded states can be found in the native basin, and the secondary structure content needs to be added as an additional dimension to properly differentiate conformations.^42^ Thus, in Figure 1F, projections are split into ranges of protein ellipticity. Panels (i), (ii), (iii), and (iv) depict the ensembles within 75–100%, 50–75%, 25–50%, and 0–25% of α-helix content, respectively. The *U*_63_^B1^ state, in panel (ii) of Figure 1F, is particularly interesting: it is a partially denatured state, characterized by the partial loss of the secondary structure of helix I of BdpA, but with mostly preserved native contacts (Supplementary Table S1 and Figures S3 and S5). The denaturation of helix I is widely recognized to be fundamental for the folding equilibrium of BdpA in water.^42,45,46^ The notable interactions of this *U*_63_^B1^ state and its destabilization in the presence of urea will be discussed in detail.

The above characterization of the SH3 and BdpA ensembles is crucial for the primary objective of this work, i.e., to thoroughly investigate urea and TMAO interactions and thermodynamic effects on the protein folding landscapes. With this aim, we obtained all-atom reconstructions of 5000 conformations from the SBM simulations, and each of these atomic models was simulated in aqueous solutions of urea or TMAO at 0.5 mol L^–1^, with constrained Cα coordinates. The solvated systems were simulated for 10 ns. For each state, as classified in Figure 1, this implied from 210 to 34130 ns of simulations (21 to 3413 structures in each subset), from which the average structure and thermodynamics of the solvents were obtained.

### Osmolyte Effects on the Folded and Unfolded States of SH3

We first focus on the effect of osmolytes urea and TMAO on the folding ensemble of the β-sheet fold of the SH3 domain, which displayed a simpler conformational landscape. Urea is a known denaturant, while TMAO is a stabilizer known to counteract the denaturing effect of urea.^17,47,48^ The structural characterization of protein–solvent interactions was possible using minimum-distance distribution functions, and the Kirkwood–Buff theory of solvation. The ComplexMixtures.jl^25^ package was developed to perform these analyses. The thermodynamics of protein–solvent interactions was characterized by measuring preferential interaction parameters, the Wyman linkage relation, and transfer free energies.^1,10,49^

MDDFs are distribution functions useful for characterizing the structure of solutions of solutes and solvents of complex shapes,^24^ as the unfolded states of proteins.^12^ MDDFs are obtained by computing the minimum-distances between any solute atom and solvent molecule atom, and thus retain a clear “solvent-shell” interpretation independently of the complexity of the structures involved. We have shown that a proper normalization of these distributions allows the practical computation of KBIs from the minimum-distance counts,^24^ thus connecting the microscopic picture of solvation to the macroscopic preferential interactions and free-energies of solvation.

Figure 2A–C display the MDDFs of water and urea relative to the protein in the native fold basin (*N*_80_^S0^) and a selected denatured state (*U*_1.0_^S12^), with 2B and 2C being insets of the most prominent peaks of these distributions. The *U*_1.0_^S12^ state was chosen for displaying the greatest preferential interactions with urea. Figure 2A shows clear density augmentation of water and urea in the protein vicinity. Both water and urea form hydrogen bonds (H-bonds) with the protein (as evidenced by the peaks at ∼1.9 Å). These H-bond peaks are greater in the native subset (Figure 2B). On the other hand, the MDDFs for the denatured state become greater at distances of nonspecific interactions (>2.5 Å). Thus, water and urea display, in average, stronger H-bonds with the native state, but stronger average nonspecific interactions with the denatured states. Both these interactions contribute to urea accumulation around the protein, and as denatured states have a greater surface area (Figure S6), a greater number of protein–solvent interactions is present. The strengthening of the nonspecific interactions upon denaturation is linked to the increased exposure of nonpolar residues (Supplementary Figure S7).

**Figure 2:**
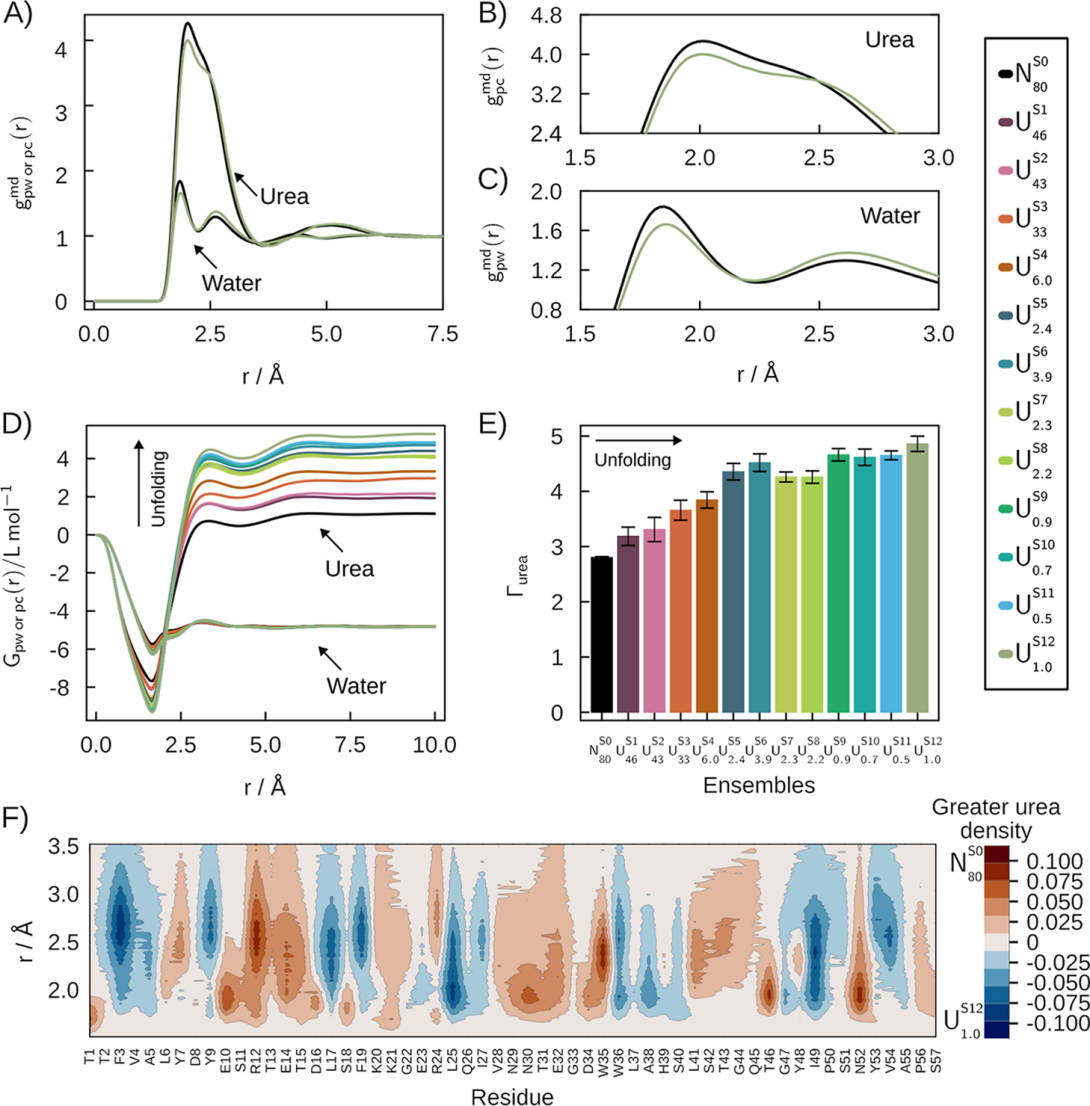
Solvation structures of the folding ensemble of the SH3 domain in 0.5 mol L^–1^ aqueous urea solutions. (A) MDDFs of water and urea for the *N*_80_^S0^ and *U*_1.0_^S12^ ensembles. (B,C) most prominent peaks in the MDDFs of urea and water. (D) KBIs and (E) Preferential interaction parameters (Γ). The preferential interactions of urea for unfolded states is greater than for the native (*N*_80_^S0^) state and increase with unfolding. (F) Difference in the breakdown of MDDF per residue in the vicinity of *N*_80_^S0^ and the unfolded *U*_1.0_^S12^ states. Red regions indicate greater urea density near the *N*_80_^S0^ state, while blue regions show higher density around the *U*_1.0_^S12^ state.

The Kirkwood–Buff Integrals (KBIs) measure the overall solvent accumulation around the protein. The KBIs for urea, in Figure 2D, are greater than those of water, implying protein dehydration, as expected.^3–6,50,51^ Thus, preferential interaction parameters with urea are always positive (Figure 2E). The distance-dependence of the KBIs, furthermore, provides insights on the contributions of interactions at each solvent shell to the buildup of the final KBIs: The initial drop (Figure 2D) is associated with the excluded protein-urea volume, which is greater for denatured states. The urea KBIs then increase sharply, outgrowing those of water, and assume roughly the final converged value at 5 Å. Therefore, the accumulation of urea at the distances of H-bonds and direct nonspecific interactions determine the protein dehydration. Quantitatively, interactions above ∼2 Å have a greater contribution to final urea KBIs than interactions at H-bonding distances (for example, for the native state, from the minimum up to ∼2 Å the KBI increases ∼2.5 L mol^–1^, and from ∼2 Å to the final KBI it increased further ∼6 L mol^–1^, a difference that increases in denatured states). The distance dependence of the KBIs depends on the choice of the integrated distance, and must be interpreted with care, but evidence supporting these strengthened nonspecific interactions with unfolded states have been obtained for urea^14^ and other denaturants.^52^

The preferential interaction parameters (Γ_urea_) roughly increase monotonically with increasing protein denaturation, being correlated with the average fraction of native contacts (*Q*), β-sheet content, and solvent accessible surface area (SASA) (Supplementary Figure S6). For instance, *N*_80_^S0^ (the native state) has the lower Γ value compared with the other ensembles: it is 1.74 times lower than the higher Γ observed in the *U*_1.0_^S12^ unfolded state. Thus, for SH3, the present results indicate that the variation of the chemical nature of the exposed surface upon denaturation does not need to be considered to qualitatively explain the urea denaturing effect, although the SASA of the side chains of nonpolar residues is consistently the most correlated with the preferential interaction parameters (Supplementary Table S2).

An in-depth molecular picture of urea accumulation at the SH3 surface in different fold states can be obtained by a differential MDDF density map (Figure 2F). This map displays the difference in the contributions of each residue to the distribution functions of urea of the *N*_80_^S0^ and *U*_1.0_^S12^ states. Red indicates a greater urea density in the native state, while blue indicates greater density in the denatured state. Urea molecules are more densely found in the first shell of the *N*_80_^S0^ state around polar residues (threonines T1 and T46, serine S18, and asparagines N30 and N52), charged glutamate E10 and arginine R12. Conversely, urea is found with greater density in the *U*_1.0_^S12^ denatured state at larger distances, around the hydrophobic residues such as phenylalanine (F3 and F19), tyrosine (Y9), leucine (L17 and L25), tryptophan (W36), isoleucine I49, and valine (V54). Similar behavior is observed for water, indicating a competition between water and urea for specific and nonspecific interactions in the folded and unfolded structures (Supplementary Figure S8). Since urea was shown to have greater affinity than water to all but charged side chains,^21^ these observations reinforce the cooperation of urea binding and the exposure of the protein core along the denaturation mechanisms. A summary of the main observations of the above analysis is provided in Table 1, which also includes the predicted transfer free energies, as will be discussed.

**Table 1:** Summary of the Main Solvation Properties for Each System^a^

	preferential solvation	notable states	key residues	δμ_p,tr_ (1M)/kcal mol^–1^
SH3 in urea	increases with unfolding	*U*_46_^S1^ (min unfolding)	+T1, −F3, -Y9, + E10, + R12, + S18, -L17, −F19, −L25, + N30, −W36, + T46, + N52, −I49, −V54	–0.47
		*U*_1.0_^S12^ (max Γ_pc_ in urea)	–F3, + E10, + R12, −L17, −F19, -L25, + W35, + T46, −I49, + N52, −V54	–1.83
SH3 in TMAO	decreases with unfolding	*U*_46_^S1^ (min unfolding)	–T1, −F3, + R12, + K20, + K21, −R24	+0.32
		*U*_1.0_^S12^ (max Γ_pc_ in urea)	–T1, −F3, −Y9, + R12, + K20, + K21, + R24, + Y48, −V54	+1.32
		*U*_0.9_^S9^ (min Γ_pc_ in TMAO)	–F3, + R12, + T13, −L17, + K20, + K21, + R24, −L25, −W36, −I49, −V54	+1.4^b^
BdpA in urea	increases with unfolding	*U*_63_^B1^ (destabilized in urea)	–F14, + E16, −K50, −L52	+0.48
		*U*_37_^B11^ (max unfolding)	+K5, + Q11, −F14, −I17, + H19, ± R28, −F31, −L35, + D37, −I45, −L46, −N53	–0.46
BdpA in TMAO	decreases with unfolding	*U*_63_^B1^ (destabilized in urea)	+K5, −K36, −K50, + K51, −K59	–0.01
		*U*_37_^B11^ (max unfolding)	+T1, + K5, + K8, −R28, + K36, −K50, + K51, + K59	+0.81

a General trend in preferential solvation; chosen states for detailed analysis; key interactions found by inspection of differential density maps (Supplementary Figures S9–S12), where + our—indicate greater or lower density in the native state; and molar transfer free energies relative to the native states at 1 mol L^–1^.

b *m*-value estimate using the additive transfer model of Auton and Bolen.^53^

Figure 3 shows the analysis of solvation structures of SH3 in aqueous solutions of TMAO. TMAO is a stabilizing osmolyte, a property associated with preferential exclusion (or, equivalently, preferential hydration).^2,54^ In Figure 3A,B we compare the MDDFs of the native state (*N*_80_^S0^—black) with the denatured state with greater TMAO exclusion (*U*_0.9_^S9^—green), and observe that the local density of TMAO in the denatured state is lower at H-bonding distances, but higher at distances associated with nonspecific interactions. Both TMAO and water, nevertheless, form specific interactions with the protein^24^ (H-bonds at ∼1.75 Å), but the TMAO peaks are much smaller than those of urea at these distances (Figure 2A,B). Also, there is a significant dip centered in ∼3.5 Å in the MDDFs of TMAO. This behavior is observed in all denatured ensembles (Supplementary Figure S13). H-bonds with the protein involve the oxygen atom of TMAO (Supplementary Figure S14A,C). The second shell of the MDDF of the TMAO arises from its amphiphilic nature: TMAO has three methyl groups, which interact favorably with the hydrophobic regions of unfolded ensembles (Supplementary Figure S14C).

**Figure 3:**
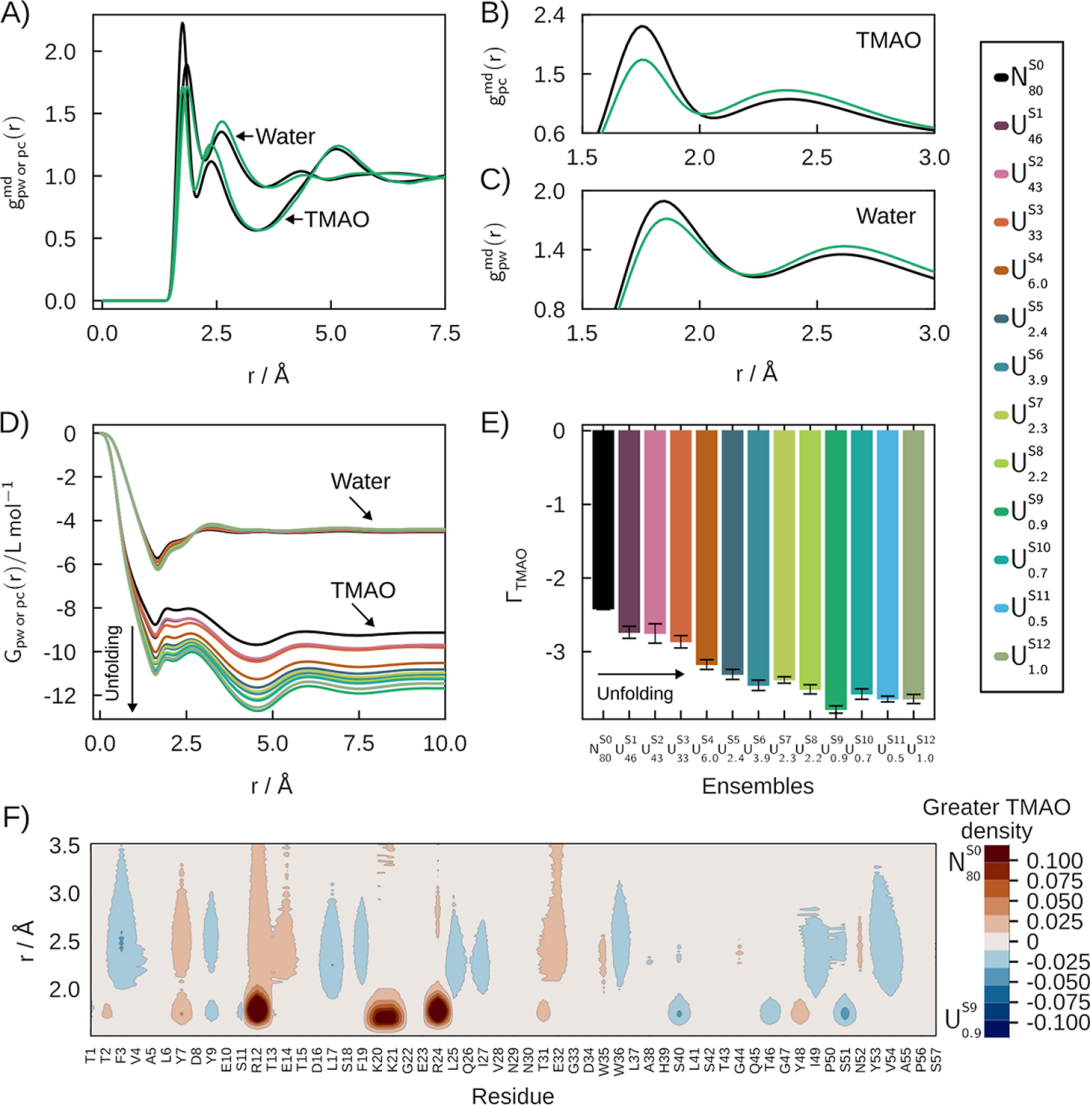
Solvation structures of the folding ensemble of the SH3 domain in 0.5 mol L^–1^ aqueous solutions of TMAO. (A) MDDFs of water and TMAO for *N*_80_^S0^ and *U*_0.9_^S9^ ensembles. (B,C) Most prominent peaks in the MDDFs of TMAO and water. (D) KBIs and (E) Preferential interaction parameters. The exclusion of TMAO for unfolded states is greater than for the native (*N*_80_^S0^) state and increases among denatured states. (F) Difference in the MDDF of the urea in the vicinity of *N*_80_^S0^ and of the unfolded *U*_0.9_^S9^ state. Red indicates greater TMAO density near the *N*_80_^S0^ state, while blue indicates higher density around the *U*_0.9_^S9^ state.

Figure 3D displays the KBIs of water and TMAO for all SH3 states, as a function of the distance. Water KBIs are similar for all ensembles, but TMAO KBIs decrease with the increasing unfolding of the protein. TMAO exclusion at short distances (<5 Å) essentially determines the converged values of the KBIs. The smaller KBIs of TMAO relative to water imply that all the ensembles exhibit preferential hydration (Γ_TMAO_ < 0) (Figure 3E), and TMAO exclusion increases with denaturation. Once again, for SH3, the detailed nature of the exposed surface is not necessary to qualitatively infer the protecting action of the cosolvent.^2,54^ On the other hand, the present analysis shows that the more denatured ensembles (*U*_6.0_^S4^ to *U*_1.0_^S12^) display greater TMAO exclusion, independently of the more hydrophobic character of the exposed protein core, which could lead to relatively greater TMAO affinity to the surface. Thus, perhaps counterintuitively, TMAO destabilization of denatured states is also cooperative.

Figure 3F shows the difference in the MDDF density of TMAO, per residue. Red and blue indicate greater TMAO density around *N*_80_^S0^ and *U*_0.9_^S9^, respectively. The density of TMAO around *N*_80_^S0^ and unfolded *U*_0.9_^S9^ are similar for most residues. Interactions in the first solvation shells of R12, R24, K20, and K21 are responsible for the greater MDDF peak at ∼1.75 Å in the native state (Figure 3B). The increase in density of the second solvation shell in the *U*_0.9_^S9^ is primarily linked with nonpolar (F3, L17, L25, W36, I49, and V54) residues (Table 1). Differential water density maps resemble those observed in the urea solutions (Supplementary Figures S8 and S15). Thus, water accumulates around the same residues independently of the cosolvent, albeit with a higher density in the TMAO solutions.

The MDDFs can be decomposed into the contributions of the backbone and side chains of the protein, as shown in Figure 4. The H-bonds responsible for the first peak of the urea MDDF have important contributions from both backbone and side chains, while for TMAO the backbone contribution is negligible. Notably, the peak associated with urea-backbone interactions becomes slightly greater for the denatured state, such that these bonds are relatively more frequent in the denatured states relative to other interactions. Interactions with the side chains are more complex: at short distances, the contributions of side chains to urea binding decreases (dashed vs solid green lines in Figure 4A), but they slightly increase for nonspecific interactions, mostly with nonpolar residues (Supplementary Figures S7 and S16A–C). The increased dehydration promoted by urea is then a combination of its greater association with the backbone at short distances, and with nonpolar residues exposed upon denaturation. TMAO preferential exclusion, on the other hand, is mostly driven by similar interaction patterns in native and denatured states, with quantitative accumulation driven by the surface area exposed in each state.

**Figure 4:**
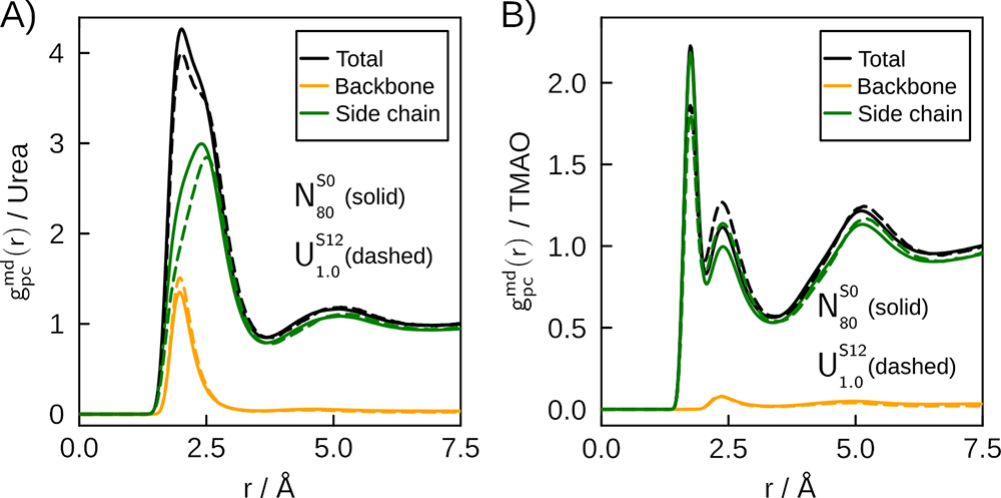
Decomposition of the MDDFs of urea and TMAO relative to SH3 in backbone and side chain contributions for the native (solid line) and a highly denatured state (dashed line).

### Osmolyte Effects on the Folded and Unfolded States of BdpA

In this section we will discuss the solvation structure of BdpA, a model α-helix protein, in the presence of urea and TMAO. On the one hand, the overall solvation properties of BdpA are similar to those of SH3: in Figure 5A,B, density augmentations of water and urea at short distances from the protein can be observed. Figure 5B shows that the H-bonding peaks are greater in the native state relative to the denatured state, but nonspecific interactions are strengthened in the denatured states. Qualitatively similar MDDF profiles are observed for TMAO in Figure 6A,B. Urea displays greater KBIs than water (Figure 5D) and solvates preferentially the BdpA domain (Figure 5E). TMAO, on the contrary, is preferentially excluded (Figure 6D,E). Urea is a better solvent for SH3 than for BdpA, and TMAO is a poorer one, as judged by the preferential interaction parameters. This is consistent with the observation that urea predominantly unfolds β-sheet structures.^55–57^

**Figure 5:**
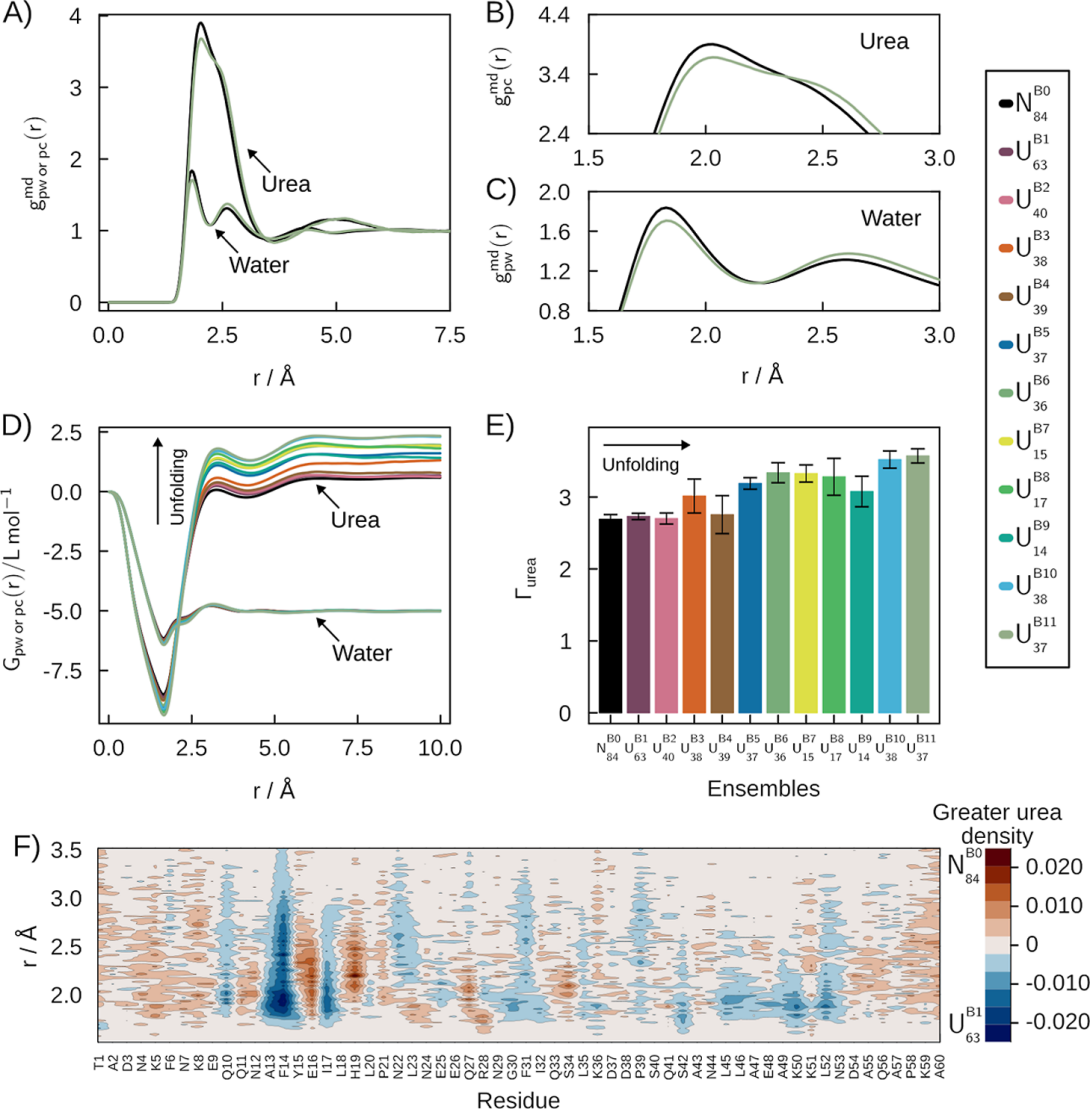
Solvation structures of the folding ensemble of BdpA in 0.5 mol L^–1^ aqueous urea solutions. (A) MDDFs of water and urea for *N*_84_^B0^ and *U*_37_^B11^ ensembles. (B,C) most prominent peaks in the MDDFs of urea and water. (D) KBIs and (E) Preferential interaction parameters. The preferential interaction of urea for unfolded states is greater than for the native (*N*_84_^B0^) state and increases among denatured states. (F) Differential density map of the MDDF breakdown per residue of *N*_84_^B0^ and *U*_63_^B1^. Red indicates greater urea density around *N*_84_^B0^ and blue around *U*_63_^B1^.

**Figure 6:**
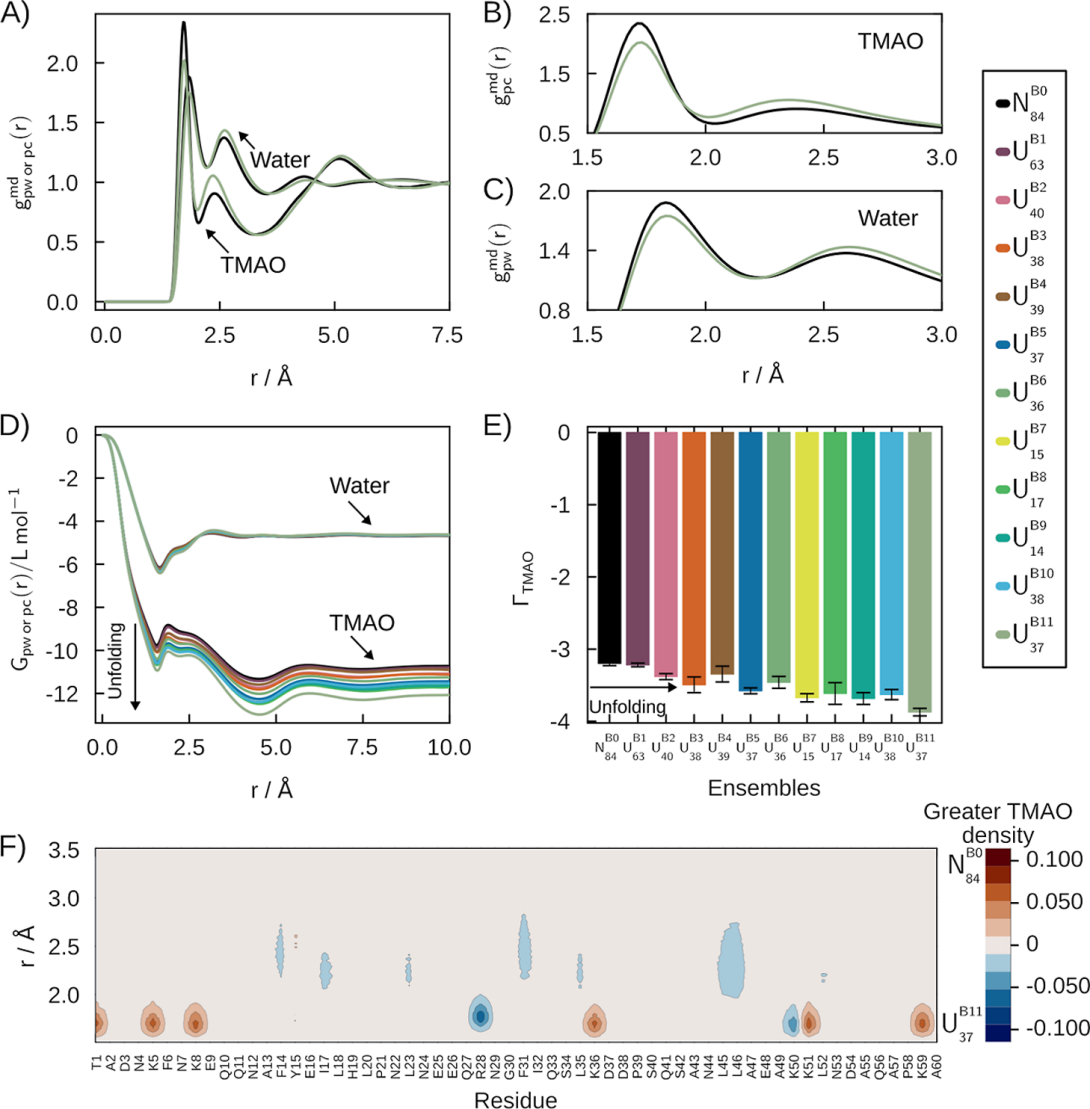
Solvation structures of BdpA in 0.5 mol L^–1^ aqueous solutions of TMAO. (A) MDDFs of water and urea for *N*_84_^B0^ and *U*_37_^B11^ ensembles. (B,C) most prominent peaks in the MDDF of TMAO and water. (D) KBIs and (E) Preferential interaction parameters. The preferential interaction of TMAO for unfolded states is lower than for the native (*N*_84_^B0^) state and decreases for denatured states. (F) Differential density map of the MDDF breakdown per residue of *N*_84_^B0^ and *U*_37_^B11^. Red indicates greater TMAO density around *N*_84_^B0^ and blue around *U*_37_^B11^.

On the other hand, what is notable about BdpA is that its first four denatured subsets, *U*_63_^B1^, *U*_40_^B2^, *U*_38_^B3^, and *U*_39_^B4^, display preferential interactions roughly equivalent to those of the native *N*_84_^B0^ state, notably in urea (Figure 5E) but also in TMAO (Figure 6E). The *U*_63_^B1^ state is, effectively, only partially denatured: it displays 63% of the crystallographic helical content and 72% of native contacts, versus 84% and 77% of the *N*_84_^B0^ ensemble from the folded basin (Supplementary Table S1). The partial denaturation of *U*_63_^B1^ is associated then to its secondary-structure compositions (Figure 1F), but not so to its surface area. *U*_63_^B1^ is an important denaturation intermediate of BdpA: it is characterized by the partial denaturation of helix I (Supplementary Figure S5). Helix I is known to be labile in BdpA, providing structural variability to the folded ensemble and leading the initial stages of denaturation.^42,45,46^ By contrast, the *U*_39_^B4^ set, with 39% of helical content and 54% of native contacts, is much further denatured. Yet, its surface accessible surface area, 52.7 nm^2^ (Supplementary Table S1) is also similar to that of the native set, of 51.2 nm^2^, indicating that it has conserved a globular fold despite the rearrangement of the secondary structure and tertiary contacts. These first four BdpA denatured sets have SASAs within 4% of that of the native ensemble, while the remaining denatured ensembles (*U*_37_^B5^ to *U*_37_^B11^) have accessible surface areas that are at least 11% greater than that of *N*_84_^B0^. Therefore, while the SASA values determine the resulting preferential interaction parameters with urea, the denatured ensemble of BdpA cannot be reduced to this parameter, and this will have important consequences to transfer free energies.

Figure 6F shows the differential density map of *U*_63_^B1^ and *N*_84_^B0^ per residue. The exposure of F14 and neighboring residue to urea highlights the link between helix I denaturation and the solvation structure. The pronounced exposure of F14 to urea is a common feature of all denatured ensembles, except of the unfolded but compact *U*_39_^B4^ (Supplementary Figure S11), where it remains buried in the protein’s hydrophobic core. Variations in the density of TMAO around each residue are smaller due to its preferential exclusion. However, in denatured states, TMAO interacts more with hydrophobic residues, and less with the charged residues, particularly lysines (Figures 6F and Supplementary S12).

In summary, BdpA interactions with urea and TMAO are qualitatively similar to those of SH3, but two sets of ensembles can be distinguished: (i) partially denatured states with a slight increase in surface area interact with the solvent similarly to the native state; (ii) denatured states with greater surface area are dehydrated by urea and preferentially hydrated in the presence of TMAO. These contrasting behaviors contribute to a multifaceted solvent dependency of the denaturation equilibrium of BdpA, which will be quantitatively discussed below.

### Osmolyte Induced Shifts in Denaturation Equilibrium Constants

The variation of the equilibrium constant, *K*, of among two folding states *U* and *N*, as a function of the osmolyte activity *a*_c_, can be obtained with the Wyman linkage equation^49,58^

1 $${\left(\frac{\partial \ln K}{\partial \ln a_{c}}\right)}_{m_{p}}=\Gamma_{pc}^{U}-\Gamma_{pc}^{N}=\Delta \Gamma_{pc}$$

where Γ are the preferential interaction parameters. ΔΓ_pc_ greater than zero implies that an increase in cosolvent concentration favors the denatured state. In qualitative terms, if the cosolvent solvates more favorably the denatured state than the native state, increasing the cosolvent concentration will shift the equilibrium toward denaturation.

Figure 7 displays ΔΓ for SH3 and BdpA in urea and TMAO at 0.5 mol L^–1^, considering the equilibrium involving the native states and each of the denatured states. The denatured states of SH3 are always stabilized by urea addition (Figure 7A—ΔΓ positive) and destabilized by TMAO (Figure 7B). For BdpA, ΔΓ is also always positive for urea, although within the modeling uncertainties for the first four partially unfolded states. Finally, TMAO favors the native state of BdpA (ΔΓ negative) relative to all denatured states, with perhaps the exception of *U*_63_^B1^. In all cases, the osmolyte effects are more prominent as the degree of denaturation increases.

**Figure 7:**
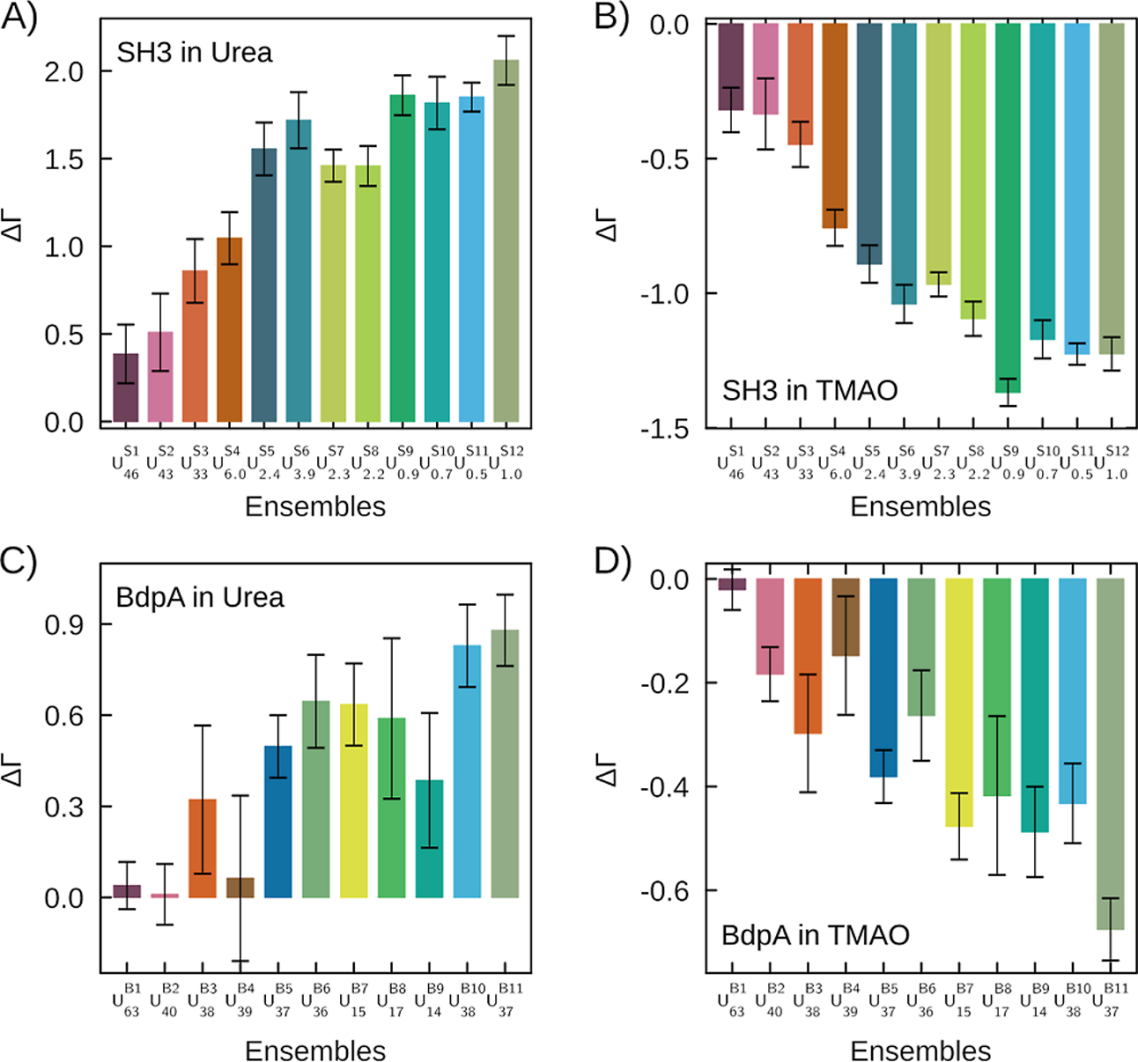
ΔΓ for each folding ensemble of structures of SH3 and BdpA proteins at osmolyte concentration of 0.5 mol L^–1^, relative to the native-state clusters. Panels (A,B) show ΔΓ values for each ensemble of SH3 in urea and TMAO. Similarly, panels (C,D) show ΔΓ values for each ensemble of BdpA.

Thus, in general, increasing the concentration of urea favors denatured states, while the native state is favored by TMAO, consistently with the overall known roles of these osmolytes on protein stability.^59^ Nevertheless, the rule breaks down for denatured states displaying similar solvent accessible surface area than the native state. The linkage relation, eq 1, does not allow, however, obtaining the absolute dependence of the equilibrium constant of denaturation (or, equivalently, on the denaturation free energy) as a function of the cosolvent concentration. It is necessary to integrate the concentration-dependence of the free energy for each state from pure water to the final ternary solution to obtain the net effect of the cosolvent on the folding equilibrium. This computation was performed for selected states, as described below.

### Osmolyte Dependence of Unfolding Free Energies for Selected Denatured States

The absolute effect of a cosolvent on the protein folding equilibrium can be determined by the difference of equilibrium constants of folding at each solvent concentration, relative to water. This corresponds to the variation of the free energy of folding upon cosolvent addition. Experimentally, these were measured by NMR with various osmolytes, including urea and TMAO,^15^ for the SH3 domain from the *Drosophila signal transduction protein* (Drk-SH3), which shares the same fold of the SH3 model here studied.^60^ Alternatively, if the folding states can be stabilized in the time-scale of the experiment, transfer free energies of each state from water to the cosolvent solution can be obtained, from which the same concentration dependence of the folding free energy can be obtained.^2^ These two approaches correspond to the reaction pairs of the Tanford Transfer thermodynamic cycle,^61,62^ shown in Figure 8.

**Figure 8:**
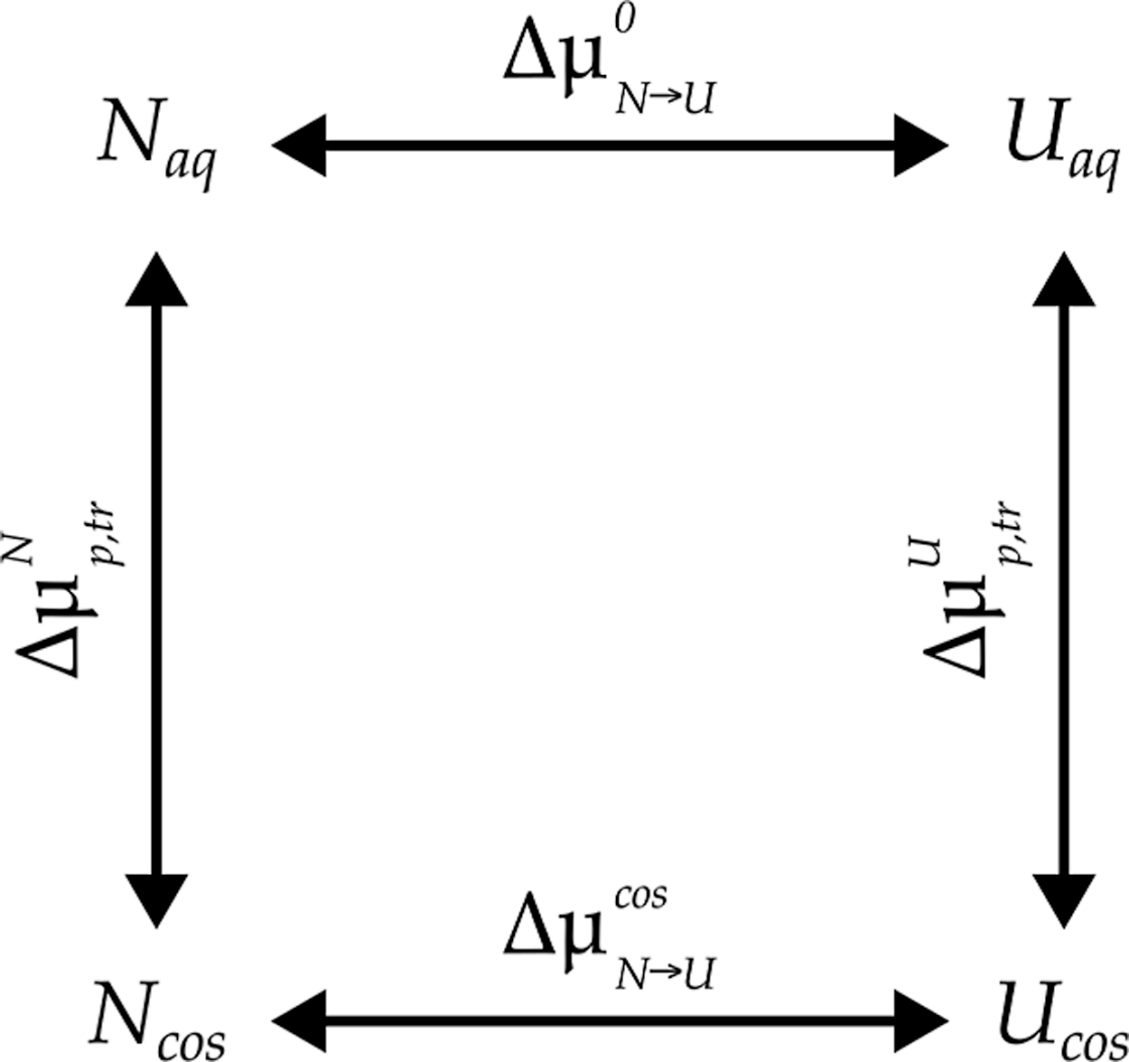
Thermodynamic cycle of the Tanford Transfer Model.^61,62^ This cycle illustrates how the folding free energies between the native (*N*) and unfolded (*U*) states in the presence of a cosolvent (*N*_cos_ ⇌ *U*_cos_, given by Δμ_N→U_^cos^) differs from that in water (*N*_aq_ ⇌ *U*_aq_, given by Δμ_N→U_^0^). Direct measures of equilibrium constants with and without cosolvent (horizontal reactions) allow the computation of the cosolvent effect on the folding free energy, ∂Δμ_p,tr_ = Δμ_N→U_^cos^ – Δμ_N→U_^0^. Alternatively, the transfer free energies for the native (Δμ_p,tr_^N^) and unfolded (Δμ_p,tr_^U^) states can be obtained (vertical reactions), and ∂Δμ_p,tr_ = Δμ_p.tr_^U^ – Δμ_p.tr_^N^.

Since computational modeling of the folding equilibrium of even small proteins such as SH3 and BdpA is difficult, the effect of the cosolvents on the folding equilibrium will be estimated by computing transfer free energies.^1,2,10,63^ The transfer free energy, Δμ_p,tr_, of a structure from pure water to a solution of the cosolvent is

2 $$\Delta \mu_{p,tr}=\int_{0}^{m_{c}}\frac{\partial \mu_{p}}{\partial m_{c}}{dm}_{c}$$

where $\frac{\partial \mu_{p}}{\partial m_{c}}$ is the variation of the chemical potential of the structure with cosolvent concentration, and the integral is performed from pure water to the desired *m*_c_ concentration of the cosolvent. This computation mimics the experiment of adding a cosolvent to an aqueous solution of a protein while roughly preserving the protein folding state, either folded or an unfolded construct.^2^

Transfer free energies can be obtained by numerical integration of eq 2, with the integrand computed from preferential interaction parameters and activities obtained at various cosolvents concentrations using^2^

3 $${\left(\frac{\partial \mu_{p}}{\partial m_{c}}\right)}_{T,P,m_{p}}=-{\left(\Gamma_{pc}\right)}_{T,P,\mu_{c}}\left(\frac{RTM_{p}}{M_{c}}\right)\left(\frac{1}{m_{c}}+\frac{\partial \ln\gamma_{c}}{\partial m_{c}}\right)$$

where γ_c_ is the cosolvent activity, and *M*_p_ and *M*_c_ are the molar masses of the protein and the cosolvent, respectively. The activities of urea and TMAO can be obtained from experimental data,^2^ and the preferential interaction parameters can be obtained by simulating a protein model in different cosolvent concentrations.

We focus on three folding states for each protein: the native states (*N*_80_^S0^ for SH3 and *N*_84_^B0^ for BdpA) and two denatured states. The denatured states were chosen from the ΔΓ extremes in urea or TMAO at 0.5 mol L^–1^ (Figure 7). We selected *U*_46_^S1^ and *U*_1.0_^S12^ for SH3, and *U*_63_^B1^ and *U*_37_^B11^ for BdpA (see Figures 1 and 2). Simulations were performed for the most representative structure of each ensemble in 7 concentrations of urea and TMAO ranging from 0.1 to 1 mol L^–1^. Physiological concentrations of these osmolytes usually fall into this range,^65,66^ although experiments can be performed at higher levels. Solution structures were characterized using MDDFs and the KB theory, as previously described, to obtain $\frac{\partial \mu_{p}}{\partial m_{c}}$ using eq 3. Preferential interactions obtained are shown in Supplementary Figure S18. The transfer free energy of each model (∂Δμ_p,tr_) was then obtained from the numerical integration of eq 2. Finally, the effect of the cosolvent in the folding free energies are δΔμ_p,tr_^U–N^ = Δμ_p,tr_^U^ – Δμ_p,tr_^N^, the difference in transfer free energies associated with the *N* ⇌ *U* transition, for each denatured state.

Figure 9A shows that the variation in unfolding free energy of SH3 states is negative for all urea concentrations, and thus the denatured states are favored. *U*_1.0_^S12^ is a highly denatured state, with a large surface area, and consistently becomes stabilized by urea to a greater extent than the more compact *U*_46_^S1^ model. In the presence of TMAO (Figure 9B), a positive ∂Δμ_p,tr_ implies destabilization of SH3 denatured states, consistently with the “osmophobic effect”.^8^ ∂Δμ_p,tr_ for *U*_46_^S1^ is smaller than for *U*_1.0_^S12^ because TMAO favors compact structures.

**Figure 9:**
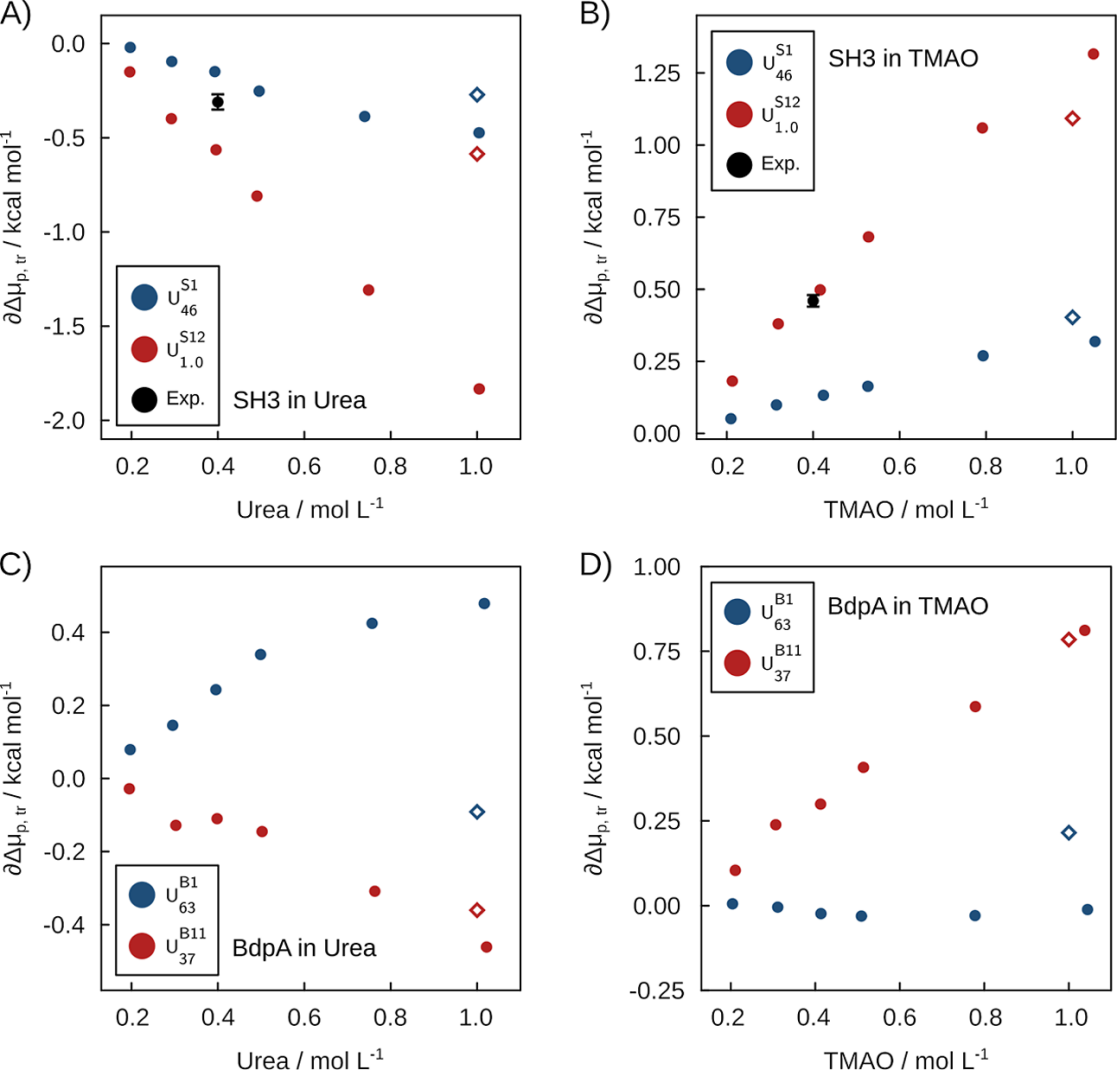
Variation of folding free energies (∂Δμ_p,tr_) relative to pure water as a function of cosolvent concentration, for selected denatured states. (A,B) ∂Δμ_p,tr_ for SH3 models in urea and TMAO, respectively. This analysis focused on the *U*_46_^S1^ (blue) and *U*_1.0_^S12^ (red) unfolded ensembles. (C,D) ∂Δμ_p,tr_ for BdpA for the folding equilibrium associated to the *U*_63_^B1^ (blue) and *U*_37_^B11^ (red) BdpA ensembles. Black circles indicate experimental data for Drk-SH3,^15^ and open diamonds correspond to SASA-based additive *m*-value estimates^53,64^ (transfer free energies at 1 mol L^–1^).

Experimentally, the free energy of Drk-SH3 folding equilibrium was shown to be affected by urea, at 0.4 mol L^–1^, by −0.31 ± 0.02 kcal mol^–1^.^15^ At this concentration, the computed variations of unfolding free energies of SH3 upon urea addition are −0.15 and −0.56 kcal mol^–1^ for the *U*_63_^S1^ and *U*_1.0_^S12^ states, respectively. Similarly, the experimentally determined variation in folding free energy in 0.4 mol L^–1^ TMAO solutions was 0.46 ± 0.02 kcal mol^–1^, and here we obtain 0.13 and 0.50 kcal mol^–1^. Experimental values are, thus, within the lower and upper bounds obtained for the effect of the cosolvents on the folding equilibria. This qualitative agreement in the sub kcal mol^–1^ energy regime is a remarkable validation of the folding ensemble obtained by the simulations, the force-fields used for representing solvent–protein interactions, and the methods used for solvent structure analysis. The experimental δΔμ_p,tr_^U–N^ are shown as black dots in Figure 9. A similar analysis of cosolvent effects on the folding equilibria of BdpA ensembles *U*_63_^B1^ and *U*_37_^B11^ is shown in Figure 9C,D. *U*_63_^B1^ has positive ∂Δμ_p,tr_^U–N^ in urea and virtually zero in the presence of TMAO. For the U_37_^B11^ state, with greater denaturation and surface area, folding free energies confirm the denaturant and protector effects of urea and TMAO (Figure 9C,D—red dots). In the previous section, we showed that the solvation characteristics of the *U*_63_^B1^ set are similar to those of the native state of BdpA. Here, we show that this is a particular case where urea exerts a destabilizing effect on a partially denatured state, while in TMAO the unfolding equilibrium involving this state is essentially independent of the cosolvent concentration. This observation was only partially deducible from the Wyman linkage relations in Figures 7C,D, considering the standard error of ΔΓ values. We cannot obtain the experimental population ratio of *U*_63_^B1^-like states vs native states from our simulations, but in our SBM model *U*_63_^B1^ was twice as populated as *N*_84_^B0^. This implies a free energy difference of roughly −0.4 kcal mol^–1^ at 300 K. The urea effect on the folding equilibrium, computed here, is in the same order of magnitude, but favoring the native state. Thus, urea could significantly reduce the population of the partially denatured helix I, redirecting unfolding along new pathways.

Transfer free energies relative to the native state at 1 mol L^–1^ (*m*-values) can be estimated by from backbone and side chain SASAs of native and denatured states and the experimental transfer free energies of the groups,^50,53,64^ and are shown as diamonds in Figure 9. The *m*-value estimates are in qualitative agreement with the computed transfer free energies, particularly in TMAO solutions where the models predict consistently the difference in stability of denatured states. For SH3 in urea (Figure 9A) the predicted *m*-values appear to underestimate the stabilization of highly denatured states and, for the BdpA, they do not capture the possible destabilization by urea of the *U*_63_^B1^ set (Figure 9C). While the absolute differences are within the accuracy of the methods employed, the empirical models are probably limited in capturing free energy variations that are a consequence of structural changes not correlated to in significant variations in solvent accessibility, which is the case of *U*_63_^B1^.

We also computed *m*-values for all equilibrated structures of SH3 and BdpA in the two solvents (Supplementary Figures S19–S21). Consistently with the visual projection of the ensemble, the denatured states of SH3 and BdpA are stabilized by urea and destabilized by TMAO. Because these models assume the additivity of the free energy, the contributions of the backbone and side chains can be obtained. In urea it is more frequent that backbone contributions are slightly greater (favoring the denatured states) than those of the side chain. In TMAO the backbones dominate the *m*-value predictions, but this was recognized as an artifact of the model.^64^

## Discussion

In this work, we developed a computational approach to characterize the effect of osmolytes on protein folding ensembles. Our pipeline combines SBMs and all-atom simulations to model the folding landscapes of SH3 and BdpA, and the solute–solvent interactions of every conformation. To elucidate the solvation structures and quantify the stabilization promoted by urea and TMAO, we employed MDDFs, which are crucial for the characterization of solvation of complex molecular shapes. We then applied the Kirkwood–Buff theory to obtain preferential interactions and the osmolyte dependence of folding free energies.

Our results confirm urea’s preferential interaction and TMAO’s preferential exclusion from protein surfaces, while adding a new dimension to how these interactions vary across the protein folding landscape. Different denatured states exhibit distinct interactions with water and cosolvents, despite significant correlations with solvent accessible surface area. Globular denatured states can be only marginally affected by osmolytes, with urea potentially promoting their destabilization. MDDFs reveal that water, urea, and TMAO form both H-bonds and nonspecific interactions with the protein surface (Figures 2A–C and 3A–C). Specific interactions are in average stronger in the native states, whereas the nonspecific interactions are strengthened in the denatured states. These patterns hold across different solvent components, protein types, and folds, reflecting the increased exposure of hydrophobic core residues upon denaturation.

Solvent exposure, fraction of native contacts, and the secondary structure elements (β-sheet and α-helix) are key determinants of preferential interactions. In the case of SH3, for which these properties are highly correlated, preferential interactions and folding free energies vary monotonically with increasing denaturation. The qualitative understanding of the unfolding paths does not depend then on the exact nature of the denatured states. On the contrary, in the case of BdpA, the secondary structure content does not directly correlate with the solvent accessible surface area or the fraction of native contacts. Thus, denatured states like *U*_63_^B1^, *U*_40_^B2^, and *U*_39_^B4^ exhibit solvation properties similar to the native state, despite their lower helical content.

Simulating representative structures of selected folding states in various TMAO and urea concentrations allowed us to compute the absolute osmolyte effect on the folding free energies. Notably, the experimentally determined folding free energy dependence on urea and TMAO for an SH3 homologous protein is found within the computed bounds, validating the force-fields, modeling strategy, and solvation analysis methods. At the same time, the solvation of a single denatured state is not sufficient to infer quantitatively the role of cosolvents in the folding equilibrium, consistently with the denatured ensemble variability playing a key role.^67^

### Limitations and Perspectives

The computational pipeline presented herein is in principle useful for the understanding of a variety of macromolecular solvation phenomena. Nevertheless, challenges remain that must be considered for each specific application. Here, we discuss some of these limitations and possible perspectives for future developments and extensions of the methods proposed.

First, a key aspect of the pipeline is the generation of the conformational ensemble. Here we opted to use SBMs which we believe provided a very broad exploration of the conformational space. However, SBMs are not parametrized for representing proteins in solvent mixtures, with two possible implications: some states that might be important in the mixture may not be sampled and, clearly, the relative populations of the states cannot be relied upon to evaluate relative stabilities. An all-atom simulation of the protein in a reference solution could be used to generate the ensemble, solving the relative population problem, but aggravating the probability of missed states. For structures without well-defined native states, as intrinsically disordered proteins or other polymers, the use of enhanced sampling simulation methods and general coarse-grained force-fields is paramount.^68–70^ Other sampling strategies can be explored, such as AI-generated unfolding ensembles^71^ or experiment-guided sampling,^68^ substituting with different trade-offs the use of SBMs. Nevertheless, by using simulations to generate ensembles, it is in principle possible to obtain the relative transfer free energies among states by integrating the solvation free energy along the transition paths. MDDFs are, at the same time, practical for understanding solvation effects across reaction paths, which can be sampled with standard enhanced sampling methods, or even if only initial and final states are available.^12,26^

Second, the quantitative (or even the qualitative) aspects of the solvation thermodynamics depends on the force-fields used. For proteins in the most common osmolyte solutions force-fields have been developed to explicitly target preferential interaction parameters obtained from experiments.^72,73^ The extension to other macromolecules and solvent mixtures depends heavily on the proper choice of the interaction parameters and may limit the applicability of the methods. Insufficient sampling of the solvent conformations can also be a problem, particularly for viscous systems, systems with strong solute–solvent or solvent–solvent correlations (requiring larger boxes and integration ranges), or computationally expensive force-fields (as polarizable models). These might require much longer equilibration and production steps for the proper convergence of KBIs and the analysis of the solution thermodynamics. Here, for instance, we have focused the majority of the analysis at 0.5 M solutions of the osmolytes because of constraints on computational resources. Transfer free energies in the 0–1 M concentration range appear to be roughly linear with concentration, allowing some generalization of the 0.5 M results, but at higher concentrations the proportionalities certainly break off.

Finally, it is important to remark the care that must be taken in interpreting causal relationships between interactions and thermodynamic outcomes. Distribution functions (MDDFs or otherwise) depend on the definition of a coordinate, which induces an interpretation of correlations between interactions and the thermodynamics of solvation. For instance, the contributions of backbones and side chains predicted by additive *m*-value (Supplementary Figure S19–S21) estimates cannot be inferred from the qualitative analysis of the MDDF decompositions in Figure 4, which show that local approximation to side chains is much more frequent than to the backbones. Different distribution functions, nevertheless, must imply the same macroscopic thermodynamics while highlighting different local interactions.

## Conclusions

Understanding the molecular mechanisms by which osmolytes modulate protein stability across the entire folding landscape, including the challenging-to-characterize denatured states, remains a critical goal in biophysics. This work addressed this challenge by employing a novel computational pipeline combining coarse-grained and all-atom simulations with minimum-distance distribution functions (MDDFs) and Kirkwood–Buff theory to dissect the thermodynamic effects of urea and TMAO on the β-sheet SH3 and α-helical BdpA domains.

We demonstrated that urea generally stabilizes unfolded states through preferential dehydration while forming strong hydrogen bonds with the protein backbones and favorable nonspecific interactions, while TMAO enhances preferential hydration, consistent with its protective effect. Crucially, these effects are conformation-dependent: urea significantly destabilizes specific partially denatured intermediates of BdpA that remain largely unaffected by TMAO, highlighting the potential for osmolytes to selectively influence folding pathways. This detailed view was enabled by the first calculation, to our knowledge, of transfer free energies for a wide range of denatured states directly from atomistic simulations, yielding folding free energies consistent with experimental data and validating our approach.

While our study focused on two specific proteins and two osmolytes, the results underscore the necessity of moving beyond native-state analyses to capture the complex interplay between solutes, cosolvents, and proteins across the conformational ensemble. The insights gained emphasize the role of nonpolar, dispersive forces in modulating solvation thermodynamics. Future work could extend this methodology to explore a broader range of osmolytes and mixtures, investigate different protein architectures, assess the influence of force field parameters, and further dissect the specific contributions of residue types to these interactions. Ultimately, this work provides a more nuanced understanding of osmolyte action and offers a robust framework for investigating macromolecular solvation in complex environments, contributing to efforts in protein engineering and solvent design.

## Methods

### Structure-Based Models (SBMs) Simulations and Analyses

Folding ensembles of the SH3 domain (PDB: 1FMK)^28^ and BdpA (PDB: 1BDD)^29^ were generated from Cα-Structure-Based Models simulations.^74,75^ The contact map of the native structures was determined using the Contact of Structural Units (CSU) algorithm,^76^ resulting in 140 and 98 contacts, respectively. The Cα-SBMs were generated using the SMOG web server (https://smog-server.org/)^77^ and simulations were performed with Gromacs 4.6.7.^78^ A simplified pipeline of the SBM simulations is shown in Supplementary Figure S24.

The folding ensemble described for BdpA in our previous publication was used in this work.^42^ A similar protocol was adopted here to obtain the SH3 folding ensemble. For the SH3 protein, the simulations were conducted in temperatures ranging from 110 to 170 units, with a temperature step of 10 units. Simulations at constant temperatures consisted of 5 × 10^8^ steps with a time step of 0.0005 reduced units. Once the temperature of maximum specific heat, *C*_v_, was roughly identified, a new set of simulations with temperatures varying between 135 and 139 with 1 unit temperature steps was performed, to localize within ∼1 unit the folding temperature (*T*_f_). In reduced temperature units, the simulations were performed within 0.91 and 1.41 with 0.083 steps and within 1.12 and 1.15 with 0.0083 temperature unit steps for the first and second sets of simulations. The simulation at the *T*_f_ was extended ten times to increase the sampling of conformational transitions.

The temperature dependence of the specific heat, *C*_v_(*T*), and the potential of mean force as a function of the fraction of native contacts, *F*(*Q*), of these simulations were obtained with WHAM,^79^ as implemented in SMOG2.^80^ A contact was classified as native if the distance between the corresponding Cα atoms did not exceed 20% of the value observed in the experimentally determined models. The statistical convergence of the *Q*-values at the *T*_f_ was confirmed by block-averaging using the MolSimToolkit.jl package (Supplementary Figure S2).

### Protein Folding Phase-Space

The energy landscape visualization method (ELViM)^35,44,81–85^ was used to represent the folding phase space of SH3 and BdpA. The ELViM method uses matrices of internal distances of conformations to establish a reliable structural similarity metric without predefined reaction coordinates. The similarity matrix is projected into a 2D space optimizing the distances between points to be correlated with the dissimilarity between structures. For this projection, we apply the Force Scheme technique,^86^ as implemented in ELViM.^35^ The secondary structures were computed with the DSSP method,^87,88^ using the ProteinSecondaryStructures.jl package.

### Atomistic Simulations and Analysis

All-atom representations of 5000 SBM models for each protein, extracted from the simulations performed at the *T*_f_ were reconstructed using the Pulchra software.^89^ We have studied the solvation of each reconstructed structure in solutions of urea 0.5 mol L^–1^ and TMAO 0.5 mol L^–1^.

Orthorhombic simulation boxes were constructed with a minimum distance of 12.0 Å from the protein extrema using Packmol,^90,91^ which creates an independent random distribution of the solvent molecules for each structure. Details of each system, including the box volume and the number of solvent molecules, are shown in Supplementary Tables S3–S6. Harmonic potentials with 25 kcal mol^–1^ force constants were applied to the Cα atoms to preserve the Cα-SBM topology during minimization, equilibration, and production, while allowing relaxation of the reconstructed atoms and solvent. The minimization and equilibration steps are necessary, in particular, to refine the all-atom reconstructed models (see Supplementary Figure S22). The CHARMM36 force field^92^ for the protein and the TIP3P water model^93^ were used. Urea molecules were modeled with the combination of CGenFF^94,95^ and the charges of the KBFF force-field, which was developed to reproduce the KBIs of urea and water molecules.^16^ TMAO was described using the Netz force field^96^ with the scaling correction proposed by Shea and co-workers,^97^ designed to accurately reproduce the Kirkwood–Buff integrals (KBI) of TMAO in ternary solutions containing water, TMAO, and ribonuclease T1 (RNase T1).^2^

All atomistic simulations were performed in Gromacs 2021.2 ^98^ at 298.15 K, 1 atm, and with a time step of 2 fs. Initially, the systems were minimized by up to 20,000 Steepest Descent steps and equilibrated for 1 ns in constant-volume and constant-temperature ensemble (*NVT*) followed by 1 ns of constant-volume and constant-pressure (*NPT*) simulation. Temperature and pressure were controlled using the modified Berendsen thermostat^99^ and Parrinello–Rahman barostat.^100^ Finally, 10 ns production simulations were performed in the *NPT* ensemble for each system, totaling 240 μs of simulation, used to obtain MDDFs, KBIs and preferential interaction parameters of Figures 2–6, and the data of Figure 7. The proper equilibration of the solvent structures was verified by the precise overlap of the MDDFs obtained from different initial random solvent distributions, and by the computation of the preferential interaction parameters using only the last 5 ns of the runs, which show a good correlation with those computed with the full 10 ns runs (Supplementary Figure S23).

Simulations in urea and TMAO at 0.1, 0.2, 0.3, 0.4, 0.5, 0.75, and 1.0 mol L^–1^, of the 6 selected representative structures of BdpA and SH3 ensembles, were performed to determine the transfer free energies used to obtain Figure 9. (see the next section for more details). Twenty replicas of 25 ns were performed for each system, totalling 42 μs of simulation (Supplementary Tables S5 and S6), to achieve a quantitative convergence of the preferential interaction parameters (Supplementary Figure S18). Representative structures were selected by the *most_representative_structure* function in MolSimToolkit.jl. The most representative structure has the minimum RMSD relative to the average structure of each set.

### Characterization of the Solvent Structure and Thermodynamics

This study employs a notation system for clarity: w (water), p (protein), c (cosolvent), and s (water or cosolvent). We developed the ComplexMixtures.jl package^25^ to characterize the solvation structures from minimum-distance distribution functions (MDDFs)^24^ and the Kirkwood–Buff theory of solvation.^101^ Specifically, the package computes the distribution functions, Kirkwood–Buff Integrals (KBIs), and atomic, group, and residue decompositions required for a molecular understanding of solvation. Density maps like that of Figure 2F were generated with the *ResidueContributions* function of ComplexMixtures.jl. From the KBIs, preferential interaction parameters (Γ) and the Wyman-linkage relation (eq 1) can be obtained.^49^ The computation of transfer free energies followed the protocol described by Lin and Timasheff.^2^ The combination of MDDFs, Γ, ΔΓ, and Δμ parameters comprehensively characterizes the solvation of the folding ensembles.

MDDFs are defined in terms of the average number density of solvent molecules *n*_c_(*r*) in the simulation, relative to the density of an ideal gas distribution *n*_c_^*^(*r*)­

4 $$MDDF\left(r\right)=\frac{n_{s}\left(r\right)}{n_{s}^{*}\left(r\right)}$$

where *r* is the minimum-distance between the protein and the cosolvent. The ideal distribution of minimum-distances has to be generated numerically to account for the shape of solute and solvent. Further discussion about MDDFs and their importance in the study of solvent structures are found in your previous publications.^24–26,102^

With an adequate reference state, the minimum-distance counts can be used to compute the Kirkwood–Buff integrals (KBIs) and preferential interaction parameters (Γs). In a finite subvolume of the system, the KBIs is given by eq 5.

5 $$G_{ps}\left(R\right)=\frac{1}{\rho_{s}}\left[N_{ps}\left(R\right)-N_{ps}^{*}\left(R\right)\right]$$

where *N*_ps_(*R*) and *N*_ps_^*^(*R*) are the number of minimum distances between the protein and the solvent within *R*, and the number of equivalent distances within *R* in an ideal system. The KBIs are the limit of *G*_ps_(*R*) for large *R*, and are the excess volume occupied by the cosolvent relative to the volume that it would occupy in the absence of solute–solvent interactions.

The thermodynamic property that measures if the cosolvent accumulates or is depleted from the protein domain, compared with the bulk solution, is the preferential interaction parameter (Γ). In the infinitely dilute solute limit, the cosolvent preferential binding to the protein (Γ_pc_) can be approximated by site counts in a finite volume defined by *R*, with

6 $$\Gamma_{pc}\approx \rho_{c}\left[G_{pc}\left(R\right)-G_{pw}\left(R\right)\right]$$

where *R* is large enough to encompass the portion of the solution affected by the presence of the solute.

Given Γ_pc_ for two states, the Wyman linkage equation^49^ (eq 1) is used to obtain the direction into which a cosolvent will drive the reaction at a given cosolvent concentration. Transfer free energies are computed using the numerical integration of eq 2, using eq 3 as the integrand, from a finite set of simulations of the states considered in different concentrations of the cosolvent. Notebooks with reproducible calculations of the transfer free energies shown in Figure 9 are available at https://m3g.github.io/PereiraMartinez2025.jl. The average of preferential interaction parameters from 20 simulations, calculated across seven cosolvent concentrations, are used to determine ∂Δμ_p,tr_, and are summarized in Supplementary Figure S18. The Plots.jl^103^ package was used to create figures.

Finally, the *m*-value estimates reported in Figure 9 were computed using the models of Moeser and Horinek^64^ for urea and Auton and Bolen^50,53^ for TMAO solutions. To compute *m*-value estimates for our denatured states we implemented the models as the *mvalue* function of MolSimToolkit.jl (v1.29.10) using the Gromacs[Bibr ref104] 2023.3 *gmx sasa* tool^105^ to compute accessible surface areas.

## Supplementary Material



## Data Availability

Data and codes necessary for the computations here reported and detailed reproduction of the computation of transfer free energies are available at https://m3g.github.io/PereiraMartinez2025.jl. For all models simulated: all-atom atom reconstructed structures, equilibrated structures, raw results from solvation analyzes of all trajectories, *m*-value estimates, solvent accessible surface area analyzes of specific residue types and chirality validation parameters are available at a Zenodo repository under DOI: 10.5281/zenodo.17155227. Additional data can be obtained upon request to authors.

## Notes

Structural characterization of folding ensembles (Table S1); correlations between preferential interaction parameters (Γ) and SASA components (Table S2); simulation box details (Tables S3 to S6); SBM folds space characterization (Figure S1 and S2); representative structure of each fold subset (Figure S3); secondary structure content per residue in each subset (Figures S4 and S5); correlation between Γ and SASA (Figure S6); MDDF decompositions of urea and TMAO by backbone and side chain contributions (Figure S7); density maps for water (Figures S8 and S15); residue contributions differential density maps for all states in urea and TMAO (Figures S9 to S12); MDDFs for all ensembles for SH3 and TMAO (Figure S13) and group contributions (Figure S14); normalized contributions of the MDDF for SH3 and BdpA proteins (S16 and S17); Γ for selected folds at multiple concentrations (Figure S18); ELViM projections of the SH3 and BdpA proteins, colored by the predicted transfer free energy (TFE) (Figure S19 and S20); decomposition of the predicted transfer free energy (ΔΔ*G*) for SH3 and BdpA protein ensembles (Figure S21); distribution of the quality of the SH3 and BdpA structures reconstructed from SBMs with Pulchra before (red) and after (blue) equilibration (Figure S22); convergence of preferential interaction parameters: 5 ns vs 10 ns (Figure S23); short SMOG pipeline used in the study (Figure S24) (PDF)
