# Supplementary material for: Osmolyte Structural and Thermodynamic Effects Across the Protein Folding Landscape
Source: JACS Au. 2025 Oct 13;5(12):6025–41. doi: 10.1021/jacsau.5c00813 (PMC12728603; doi:10.1021/jacsau.5c00813)
Supplement: Supplementary file 1 [file au5c00813_si_001.pdf]

Supplementary Information

# Osmolyte structural and thermodynamic effects across the protein folding landscape

*Ander Francisco Pereira and Leandro Martínez**

Institute of Chemistry and Center for Computing in Engineering & Science, Universidade Estadual de Campinas (UNICAMP), 13083-861 Campinas, SP, Brazil

**Contents:**

1. Structural characterization of folding ensembles (Table S1)
2. Correlations between preferential interaction parameters (Γ) and SASA components (Table S2)
3. Simulation box details (Tables S3 to S6).
4. SBM folds space characterization (Figures S1 and S2)
5. Representative structure of each fold subset (Figure S3)
6. Secondary structure content per residue in each subset (Figures S4 and S5)
7. Correlation between Γ and SASA (Figure S6)
8. MDDF decompositions of urea and TMAO by backbone and side chain contributions (Figure S7)
9. Density maps for water (Figures S8 and S15)
10. Residue contributions differential density maps for all states in urea and TMAO (Figures S9 to S12)
11. MDDFs for all ensembles for SH3 and TMAO (Figure S13) and group contributions (Figure S14)
12. Normalized contributions of the MDDF for SH3 and BdpA proteins (S16 and S17)

13. Γ for selected folds at multiple concentrations (Figure S18)
14. ELViM projections of the SH3 and BdpA proteins, colored by the predicted transfer free energy (TFE) (Figures S19 and S20)
15. Decomposition of the predicted transfer free energy (ΔΔG) for SH3 and BdpA protein ensembles (Figure S21)
16. Distribution of the quality of the SH3 and BdpA structures reconstructed from SBMs with Pulchra before (red) and after (blue) equilibration (Figure S22)
17. Convergence of preferential interaction parameters: 5 ns vs. 10 ns (Figure S23)
18. Short SMOG pipeline used in the study (Figure S24)

**Table S1**. Fraction of native contacts (Q), Solvent Accessible Surface Area (SASA), secondary structure content (β-sheet (SH3) and $\alpha$-helix (BdpA)), preferential interaction parameters of Urea and TMAO for each ensemble of the SH3 and BdpA proteins.

SH3 protein

| Ensembles | Average Q | Average SASA ($nm^2$) | Average β-sheet content | Average $\Gamma_{TMAO}$ | Average $\Gamma_{Urea}$ |
|---|---|---|---|---|---|
| $N_{80}^{S0}$ | 0.850 | 42.413 | 80.018 | -2.416 | 2.800 |
| $U_{46}^{S1}$ | 0.553 | 48.828 | 45.584 | -2.737 | 3.185 |
| $U_{43}^{S2}$ | 0.552 | 47.512 | 42.650 | -2.752 | 3.309 |
| $U_{33}^{S3}$ | 0.544 | 47.831 | 33.044 | -2.865 | 3.659 |
| $U_{6.0}^{S4}$ | 0.236 | 56.175 | 5.999 | -3.175 | 3.846 |
| $U_{2.4}^{S5}$ | 0.176 | 58.718 | 2.437 | -3.309 | 4.355 |
| $U_{3.9}^{S6}$ | 0.170 | 62.640 | 3.932 | -3.464 | 4.519 |
| $U_{2.3}^{S7}$ | 0.159 | 62.128 | 2.258 | -3.391 | 4.260 |
| $U_{2.2}^{S8}$ | 0.139 | 65.246 | 2.220 | -3.509 | 4.258 |
| $U_{0.9}^{S9}$ | 0.106 | 67.949 | 0.892 | -3.783 | 4.661 |
| $U_{0.7}^{S10}$ | 0.097 | 66.900 | 0.689 | -3.575 | 4.617 |
| $U_{0.5}^{S11}$ | 0.091 | 68.472 | 0.502 | -3.645 | 4.651 |
| $U_{1.0}^{S12}$ | 0.087 | 69.213 | 1.040 | -3.642 | 4.860 |

BdpA protein

| Ensembles | Average Q | Average SASA ($nm^2$) | Average $\alpha$-helix content | Average $\Gamma_{TMAO}$ | Average $\Gamma_{Urea}$ |
|---|---|---|---|---|---|
| $N_{84}^{B0}$ | 0.772 | 51.248 | 84.495 | -3.198 | 2.685 |
| $U_{63}^{B1}$ | 0.729 | 52.156 | 62.579 | -3.219 | 2.731 |
| $U_{40}^{B2}$ | 0.609 | 53.069 | 40.224 | -3.382 | 2.702 |
| $U_{38}^{B3}$ | 0.553 | 53.303 | 37.951 | -3.496 | 3.014 |
| $U_{39}^{B4}$ | 0.543 | 52.673 | 38.889 | -3.346 | 2.755 |
| $U_{37}^{B5}$ | 0.378 | 58.953 | 36.962 | -3.579 | 3.189 |
| $U_{36}^{B6}$ | 0.370 | 57.210 | 35.996 | -3.461 | 3.337 |
| $U_{15}^{B7}$ | 0.348 | 59.481 | 15.067 | -3.675 | 3.327 |
| $U_{17}^{B8}$ | 0.336 | 61.114 | 17.224 | -3.616 | 3.281 |
| $U_{14}^{B9}$ | 0.335 | 59.255 | 13.642 | -3.685 | 3.077 |
| $U_{38}^{B10}$ | 0.333 | 59.279 | 37.873 | -3.631 | 3.520 |
| $U_{37}^{B11}$ | 0.305 | 64.460 | 36.861 | -3.874 | 3.571 |

**Table S2**. Correlations between preferential interaction parameters (Γ) and SASA components for SH3 and BdpA in urea and TMAO.

| Protein | Correlation | $R^2$ |
|---|---|---|
| SH3 | $\Gamma_{urea}$ vs SASA (total) | 0.93 |
| SH3 | $\Gamma_{urea}$ vs SC_Nonpolar | 0.93 |
| SH3 | $\Gamma_{urea}$ vs SC_PolarUncharged | 0.91 |
| SH3 | $\Gamma_{urea}$ vs SC_PolarCharged | 0.73 |
| SH3 | $\Gamma_{urea}$ vs BB_Nonpolar | 0.93 |
| SH3 | $\Gamma_{urea}$ vs BB_PolarUncharged | 0.94 |
| SH3 | $\Gamma_{urea}$ vs BB_PolarCharged | 0.91 |
| SH3 | $\Gamma_{TMAO}$ vs SASA (total) | 0.97 |
| SH3 | $\Gamma_{TMAO}$ vs SC_Nonpolar | 0.98 |
| SH3 | $\Gamma_{TMAO}$ vs SC_PolarUncharged | 0.95 |
| SH3 | $\Gamma_{TMAO}$ vs SC_PolarCharged | 0.83 |
| SH3 | $\Gamma_{TMAO}$ vs BB_Nonpolar | 0.98 |
| SH3 | $\Gamma_{TMAO}$ vs BB_PolarUncharged | 0.98 |
| SH3 | $\Gamma_{TMAO}$ vs BB_PolarCharged | 0.96 |
| BdpA | $\Gamma_{urea}$ vs SASA (total) | 0.81 |
| BdpA | $\Gamma_{urea}$ vs SC_Nonpolar | 0.85 |
| BdpA | $\Gamma_{urea}$ vs SC_PolarUncharged | 0.58 |
| BdpA | $\Gamma_{urea}$ vs SC_PolarCharged | 0.20 |
| BdpA | $\Gamma_{urea}$ vs BB_Nonpolar | 0.69 |
| BdpA | $\Gamma_{urea}$ vs BB_PolarUncharged | 0.63 |
| BdpA | $\Gamma_{urea}$ vs BB_PolarCharged | 0.43 |
| BdpA | $\Gamma_{TMAO}$ vs SASA (total) | 0.88 |
| BdpA | $\Gamma_{TMAO}$ vs SC_Nonpolar | 0.90 |
| BdpA | $\Gamma_{TMAO}$ vs SC_PolarUncharged | 0.59 |
| BdpA | $\Gamma_{TMAO}$ vs SC_PolarCharged | 0.20 |
| BdpA | $\Gamma_{TMAO}$ vs BB_Nonpolar | 0.89 |
| BdpA | $\Gamma_{TMAO}$ vs BB_PolarUncharged | 0.87 |
| BdpA | $\Gamma_{TMAO}$ vs BB_PolarCharged | 0.76 |

**Table S3**. Simulation box details for each SH3 protein ensemble in 0.5 mol $L^{-1}$ urea and TMAO solutions.

| SH3 protein | | | |
|---|---|---|---|
| Ensembles | Average Box Volume (nm3) and SE | Average Water Number and SE | Average Cosolvent Number and SE |
| $N_{80}^{S0}$ | 162.1 ± 0.2 | 4576 ± 6 | 42.78 ± 0.05 |
| $U_{46}^{S1}$ | 182 ± 3 | 5207 ± 86 | 48.7 ± 0.8 |
| $U_{43}^{S2}$ | 180 ± 3 | 5132 ± 82 | 48.0 ± 0.8 |
| $U_{33}^{S3}$ | 177 ± 3 | 5035 ± 86 | 47.1 ± 0.8 |
| $U_{6.0}^{S4}$ | 207 ± 2 | 5957 ± 78 | 55.7 ± 0.7 |
| $U_{2.4}^{S5}$ | 228 ± 3 | 6616 ± 108 | 62 ± 1 |
| $U_{3.9}^{S6}$ | 250 ± 3 | 7296 ± 101 | 68.2 ± 0.9 |
| $U_{2.3}^{S7}$ | 249 ± 3 | 7268 ± 82 | 67.9 ± 0.8 |
| $U_{2.2}^{S8}$ | 284 ± 4 | 8353 ± 129 | 78 ± 1 |
| $U_{0.9}^{S9}$ | 330 ± 7 | 9806 ± 234 | 92 ± 2 |
| $U_{0.7}^{S10}$ | 282 ± 4 | 8286 ± 113 | 78 ± 1 |
| $U_{0.5}^{S11}$ | 322 ± 4 | 9549 ± 112 | 89 ± 1 |
| $U_{1.0}^{S12}$ | 313 ± 4 | 9268 ± 124 | 87 ± 1 |

**Table S4**. Simulation box details for each BdpA protein ensemble in 0.5 mol $L^{-1}$ urea and TMAO solutions.

| BdpA protein | | | |
|---|---|---|---|
| Ensembles | Average Box Volume (nm3) and SE | Average Water Number and SE | Average Cosolvent Number and SE |
| $N_{84}^{B0}$ | 208.1 ± 0.9 | 5985 ± 28 | 55.9 ± 0.3 |
| $U_{63}^{B1}$ | 207.1 ± 0.8 | 5953 ± 23 | 55.7 ± 0.2 |
| $U_{40}^{B2}$ | 203 ± 1 | 5822 ± 37 | 54.4 ± 0.3 |
| $U_{38}^{B3}$ | 191 ± 2 | 5467 ± 68 | 51.1 ± 0.6 |
| $U_{39}^{B4}$ | 192 ± 3 | 5496 ± 94 | 51.2 ± 0.9 |
| $U_{37}^{B5}$ | 242 ± 2 | 7023 ± 55 | 65.7 ± 0.5 |
| $U_{36}^{B6}$ | 221 ± 2 | 6392 ± 72 | 59.8 ± 0.7 |
| $U_{15}^{B7}$ | 235 ± 2 | 6818 ± 74 | 63.7 ± 0.7 |
| $U_{17}^{B8}$ | 251 ± 4 | 7324 ± 120 | 68 ± 1 |
| $U_{14}^{B9}$ | 223 ± 3 | 6446 ± 104 | 60 ± 1 |
| $U_{38}^{B10}$ | 240 ± 4 | 6973 ± 110 | 65 ± 1 |
| $U_{37}^{B11}$ | 302 ± 4 | 8909 ± 105 | 83 ± 1 |

**Table S5**. Simulation box details for each representative SH3 protein structure in 0.1, 0.2, 0.3, 0.4, 0.5, 0.75, and 1.0 mol L$^{-1}$ urea and TMAO solutions.

| SH3 protein | | | | |
|---|---|---|---|---|
| Representative structure | Concentration (mol L$^{-1}$) | Box Volume (nm$^3$) | Water Number | Cosolvent Number |
| $N_{80}^{S0}$ | 0.1 | 228.54 | 6826 | 12 |
| $N_{80}^{S0}$ | 0.2 | 227.73 | 6772 | 25 |
| $N_{80}^{S0}$ | 0.3 | 227.79 | 6722 | 37 |
| $N_{80}^{S0}$ | 0.4 | 227.60 | 6667 | 50 |
| $N_{80}^{S0}$ | 0.5 | 227.71 | 6617 | 62 |
| $N_{80}^{S0}$ | 0.75 | 228.00 | 6488 | 93 |
| $N_{80}^{S0}$ | 1 | 228.11 | 6359 | 124 |
| $U_{46}^{S1}$ | 0.1 | 254.66 | 7674 | 14 |
| $U_{46}^{S1}$ | 0.2 | 255.27 | 7615 | 28 |
| $U_{46}^{S1}$ | 0.3 | 254.78 | 7557 | 42 |
| $U_{46}^{S1}$ | 0.4 | 255.14 | 7499 | 56 |
| $U_{46}^{S1}$ | 0.5 | 255.04 | 7440 | 70 |
| $U_{46}^{S1}$ | 0.75 | 255.39 | 7298 | 104 |
| $U_{46}^{S1}$ | 1 | 254.65 | 7152 | 139 |
| $U_{1.0}^{S12}$ | 0.1 | 322.87 | 9851 | 18 |
| $U_{1.0}^{S12}$ | 0.2 | 322.07 | 9776 | 36 |
| $U_{1.0}^{S12}$ | 0.3 | 323.07 | 9701 | 54 |
| $U_{1.0}^{S12}$ | 0.4 | 322.02 | 9630 | 71 |
| $U_{1.0}^{S12}$ | 0.5 | 322.17 | 9555 | 89 |
| $U_{1.0}^{S12}$ | 0.75 | 322.43 | 9367 | 134 |
| $U_{1.0}^{S12}$ | 1 | 322.49 | 9179 | 179 |

**Table S6**. Simulation box details for each representative BdpA protein structure in 0.1, 0.2, 0.3, 0.4, 0.5, 0.75, and 1.0 mol L$^{-1}$ urea and TMAO solutions.

| BdpA protein | | | | |
|---|---|---|---|---|
| Representative structure | Concentration (mol L$^{-1}$) | Box Volume (nm$^3$) | Water Number | Cosolvent Number |
| $N_{84}^{B0}$ | 0.1 | 327.229 | 10000 | 18 |
| $N_{84}^{B0}$ | 0.2 | 327.344 | 9924 | 36 |
| $N_{84}^{B0}$ | 0.3 | 327.15 | 9849 | 54 |
| $N_{84}^{B0}$ | 0.4 | 327.72 | 9770 | 73 |
| $N_{84}^{B0}$ | 0.5 | 327.294 | 9695 | 91 |
| $N_{84}^{B0}$ | 0.75 | 327.065 | 9507 | 136 |
| $N_{84}^{B0}$ | 1 | 327.14 | 9319 | 181 |
| $U_{63}^{B1}$ | 0.1 | 305.938 | 9262 | 17 |
| $U_{63}^{B1}$ | 0.2 | 306.058 | 9191 | 34 |
| $U_{63}^{B1}$ | 0.3 | 306.046 | 9124 | 50 |
| $U_{63}^{B1}$ | 0.4 | 305.645 | 9053 | 67 |
| $U_{63}^{B1}$ | 0.5 | 306.011 | 8983 | 84 |
| $U_{63}^{B1}$ | 0.75 | 306.166 | 8807 | 126 |
| $U_{63}^{B1}$ | 1 | 305.406 | 8632 | 168 |
| $U_{37}^{B11}$ | 0.1 | 396.974 | 11673 | 21 |
| $U_{37}^{B11}$ | 0.2 | 397.009 | 11585 | 42 |
| $U_{37}^{B11}$ | 0.3 | 396.789 | 11493 | 64 |
| $U_{37}^{B11}$ | 0.4 | 398.107 | 11406 | 85 |
| $U_{37}^{B11}$ | 0.5 | 396.861 | 11318 | 106 |
| $U_{37}^{B11}$ | 0.75 | 397.379 | 11097 | 159 |
| $U_{37}^{B11}$ | 1 | 397.272 | 10876 | 212 |

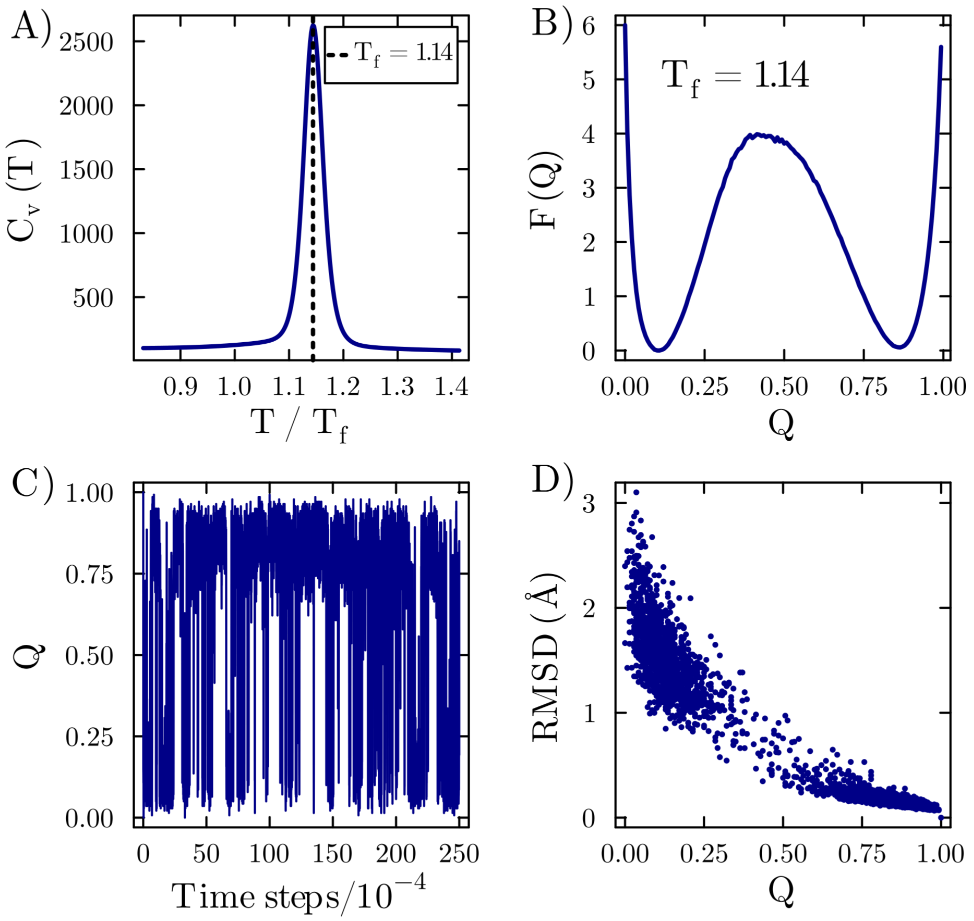


**Figure S1**. Characterization of SH3 domain folding. A) Specific heat ($C_v$) as a function of temperature, allowing the folding temperature identification ($T_f$ = 0.97 reduced units). From the simulation performed at the $T_f$: B) Free energy as a function of the fraction of native contacts (Q). C) Fraction of native contacts (Q) as a function of the simulation time step. D) Contour maps of the Probability Density (PD) as a function of Q and RMSD.

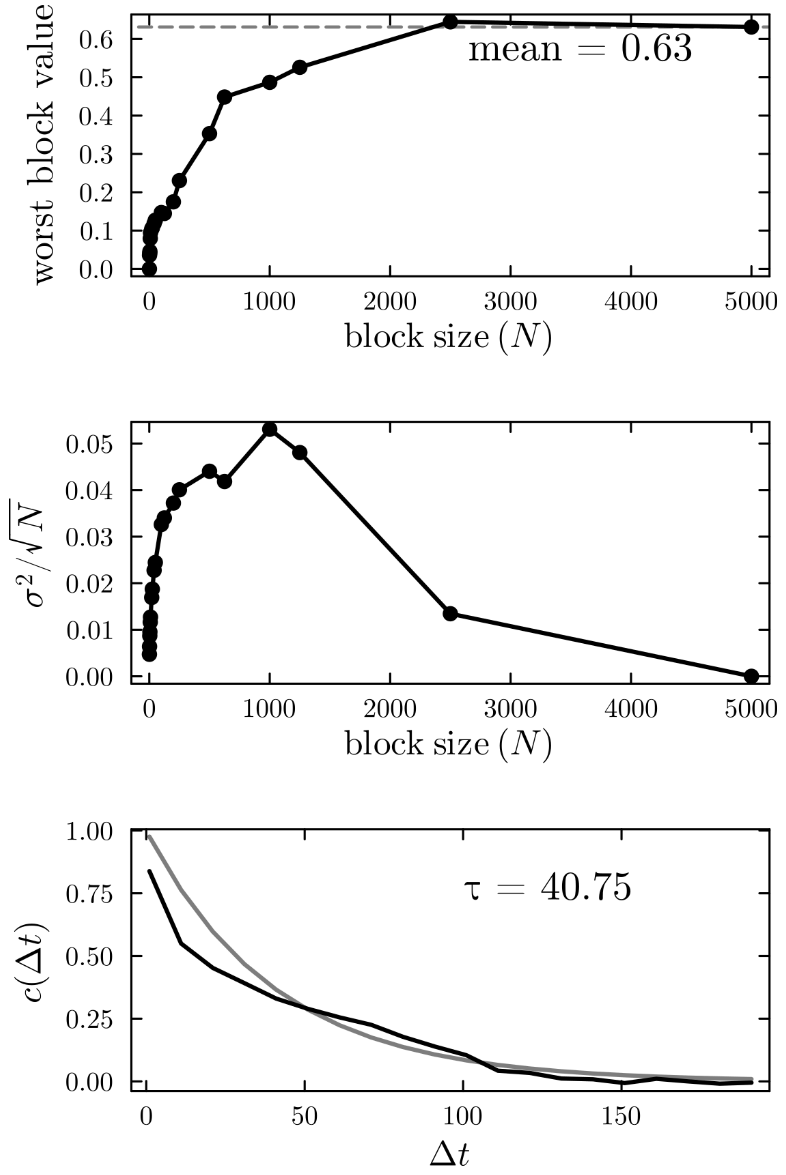


**Figure S2**. Block-averaging and autocorrelation function for the fraction of native contacts (Q) derived from simulation with $C_\alpha$-SBMs at the folding temperature ($T_f$). The analysis employed the block_average function of the MolSimToolkit.jl package, accessible at http://github.com/m3g/MolSimToolkit.jl/ - version 1.3.4. The presented data distinctly show simulation convergence, as the block average value rapidly approaches the average value of the property (Q), the standard deviation is low, and the correlation time decays quickly.

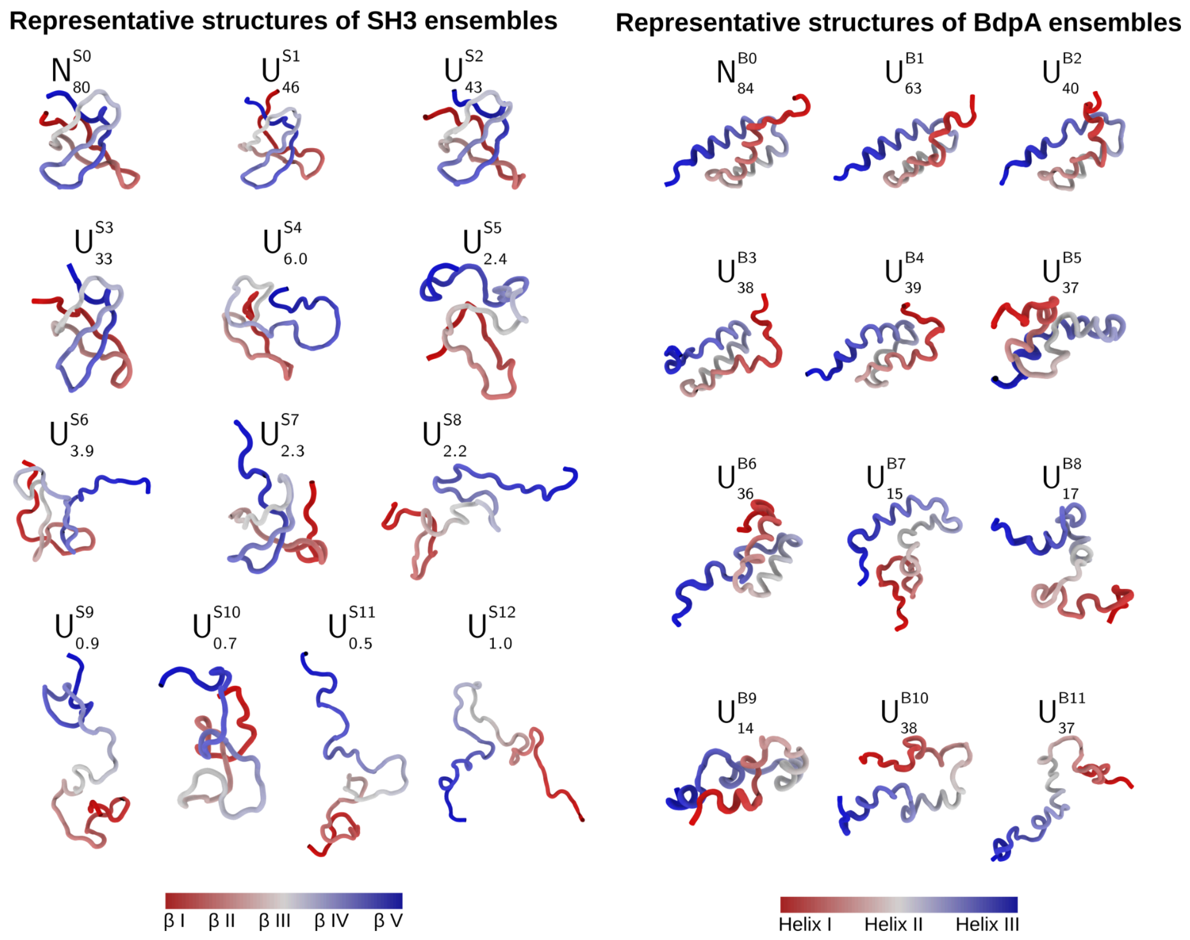


**Figure S3**. Representative structures of SH3 and BdpA ensembles.

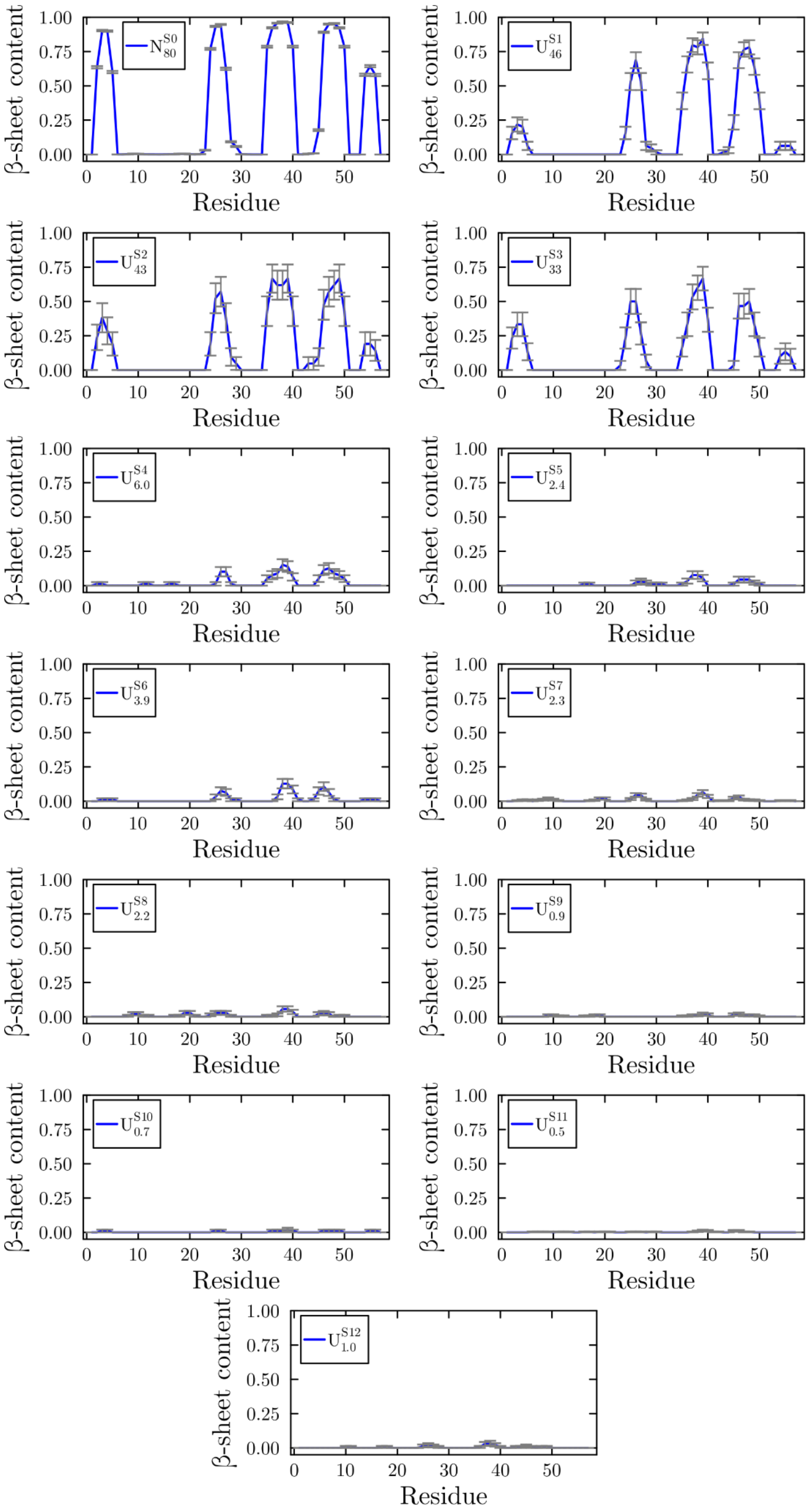


**Figure S4**. $\beta$-sheet content per residue in each SH3 folding subset.

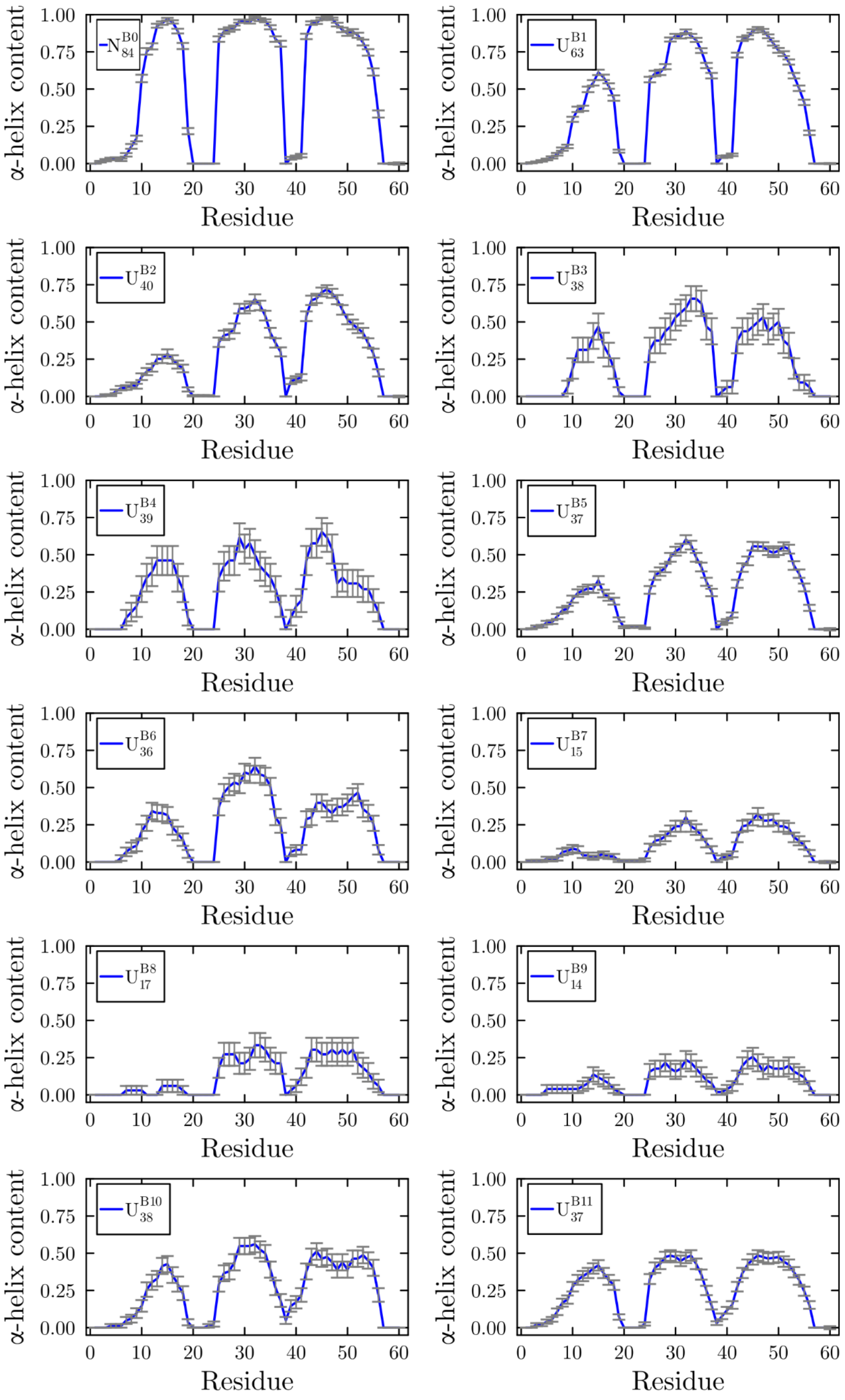


**Figure S5**. Helical content per residue in each BdpA folding subset.

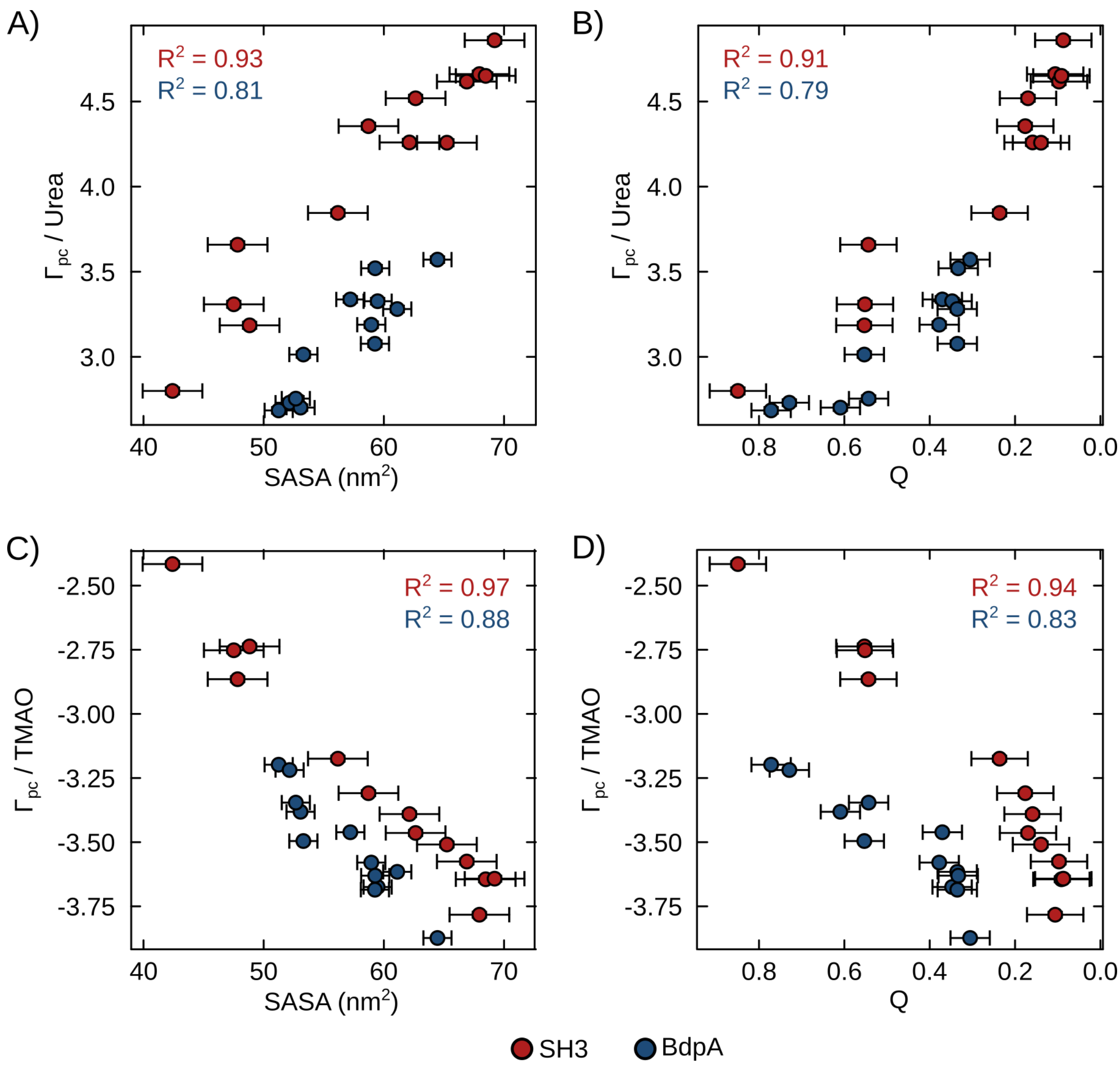


**Figure S6**. Preferential integration parameters (Γs) as a function of SASA (A) and C)) and Q (B) and D)). Red and blue dots represent the average parameters for SH3 and BdpA protein ensembles, respectively, with corresponding error bars. A linear fit effectively captures the correlation between Γs and SASA or Q in each case. For SH3, Q is strongly correlated with both SASA ($R^2$ = 0.92) and β-sheet content ($R^2$ = 0.97). For BdpA, Q also shows strong correlations with SASA ($R^2$ = 0.83) and $\alpha$-helix content (R = 0.69).

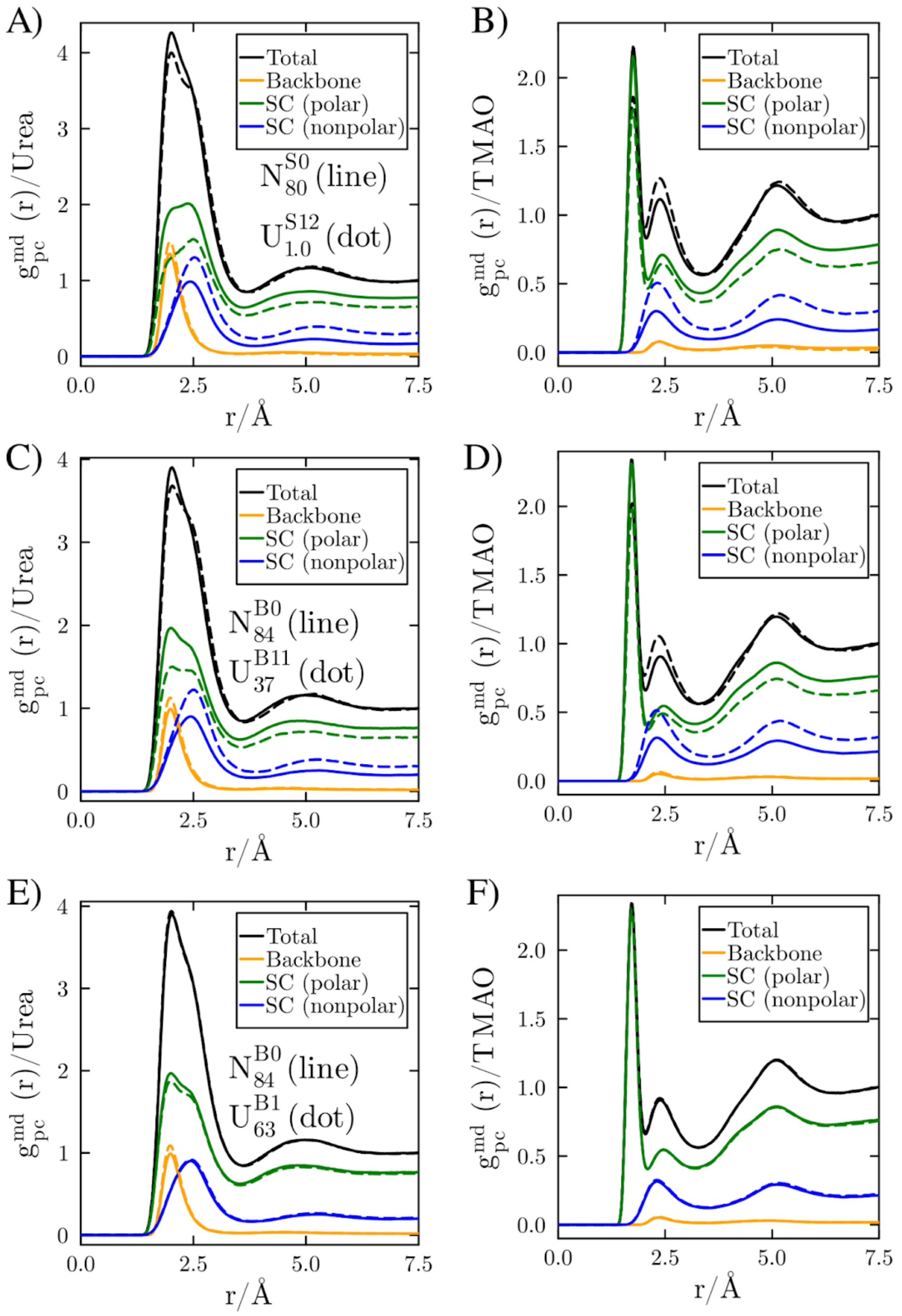


**Figure S7**. Decomposition of the MDDFs of urea (panels A, C, and E) and TMAO (panels B, D, and F) into contributions from the backbone (orange), polar side chain (green), and nonpolar side chain (blue). Solid lines represent the native ensembles, and dashed lines represent the denatured ensembles. Panels A and B compare the SH3 domain ensembles $N_{80}^{S0}$ and $U_{1.0}^{S12}$ in urea and TMAO, respectively. Panels C and D show MDDFs for BdpA, comparing ensembles $N_{84}^{B0}$ and $U_{37}^{B11}$ in urea and TMAO, respectively. Panels E and F show the same comparison as in C and D, but with a partially denatured ensemble $U_{63}^{B1}$.

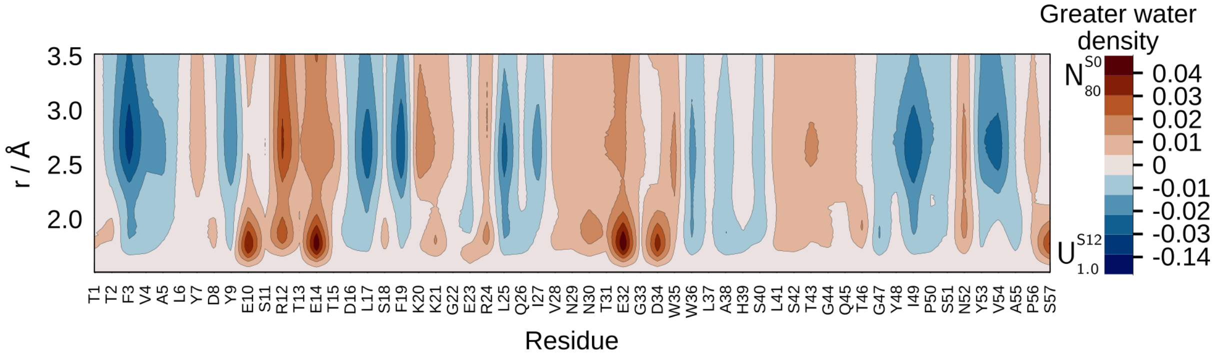


**Figure S8**. Difference in the MDDF density of the water in the vicinity of $N_{80}^{S0}$ and unfolded ($U_{1.0}^{S12}$) state in the urea 0.5 mol L$^{-1}$ solution. Red regions indicate higher water density near the $N_{80}^{S0}$ state, while blue regions show higher density around the $U_{1.0}^{S12}$ state. This pattern emphasizes the increased interactions of the solvent with mostly hydrophobic residues in the $U_{1.0}^{S12}$ state that are typically protected from the solvent in the $N_{80}^{S0}$ conformation.

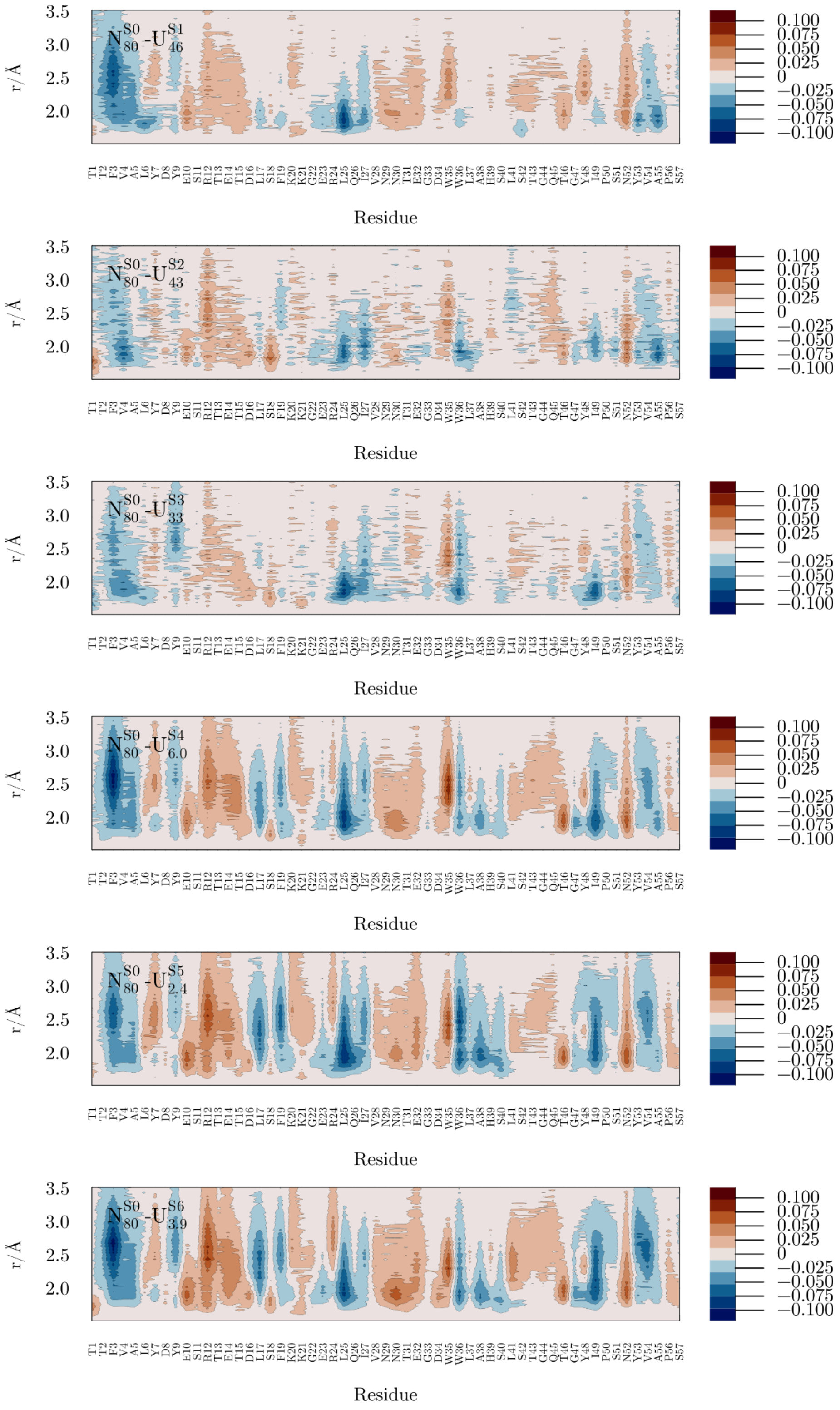


**Figure S9**. Differential density maps per residue for SH3 in urea solution 0.5 mol L$^{-1}$.

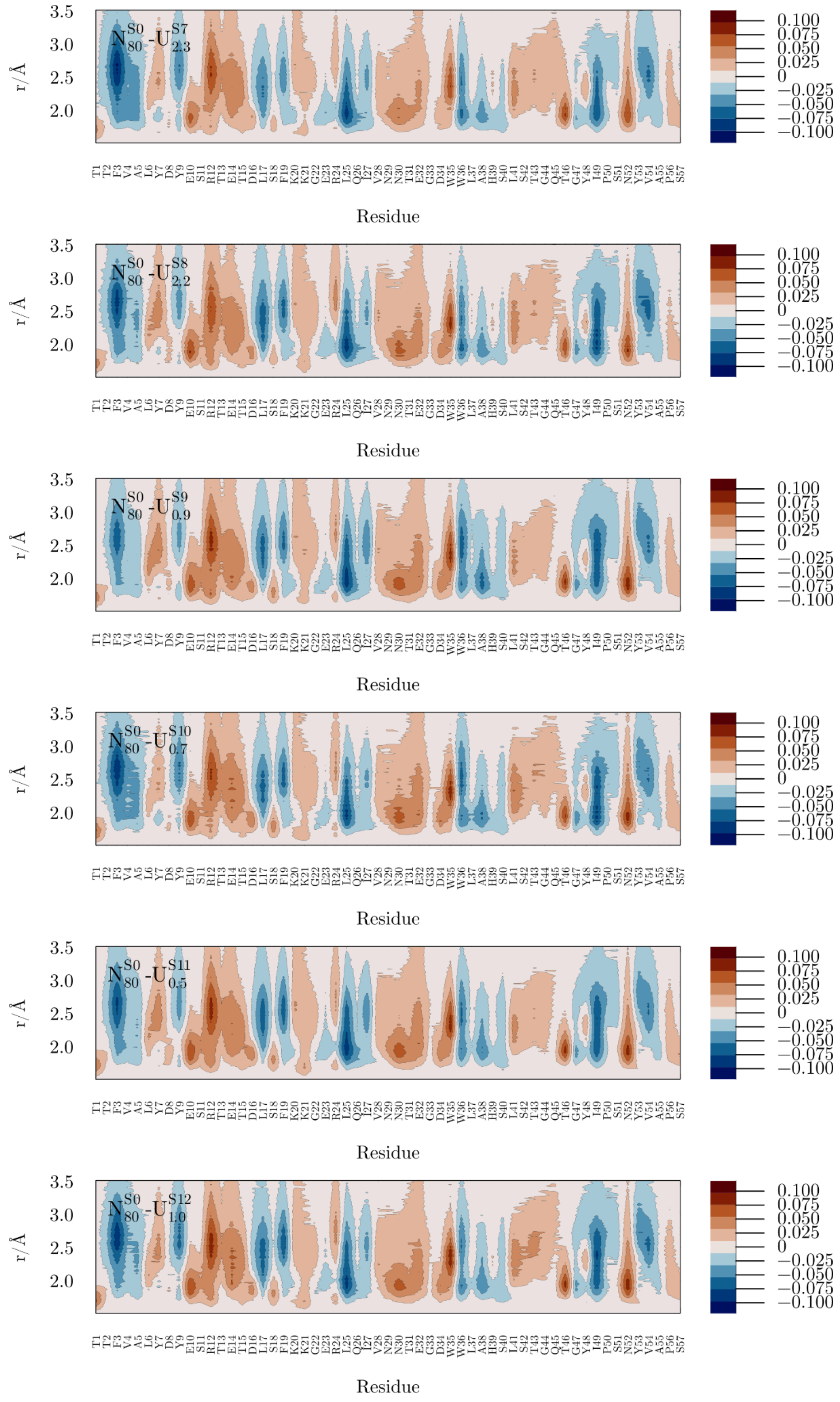


**Figure S9 (continued)**. Differential density maps per residue for SH3 in urea solution 0.5 mol $L^{-1}$.

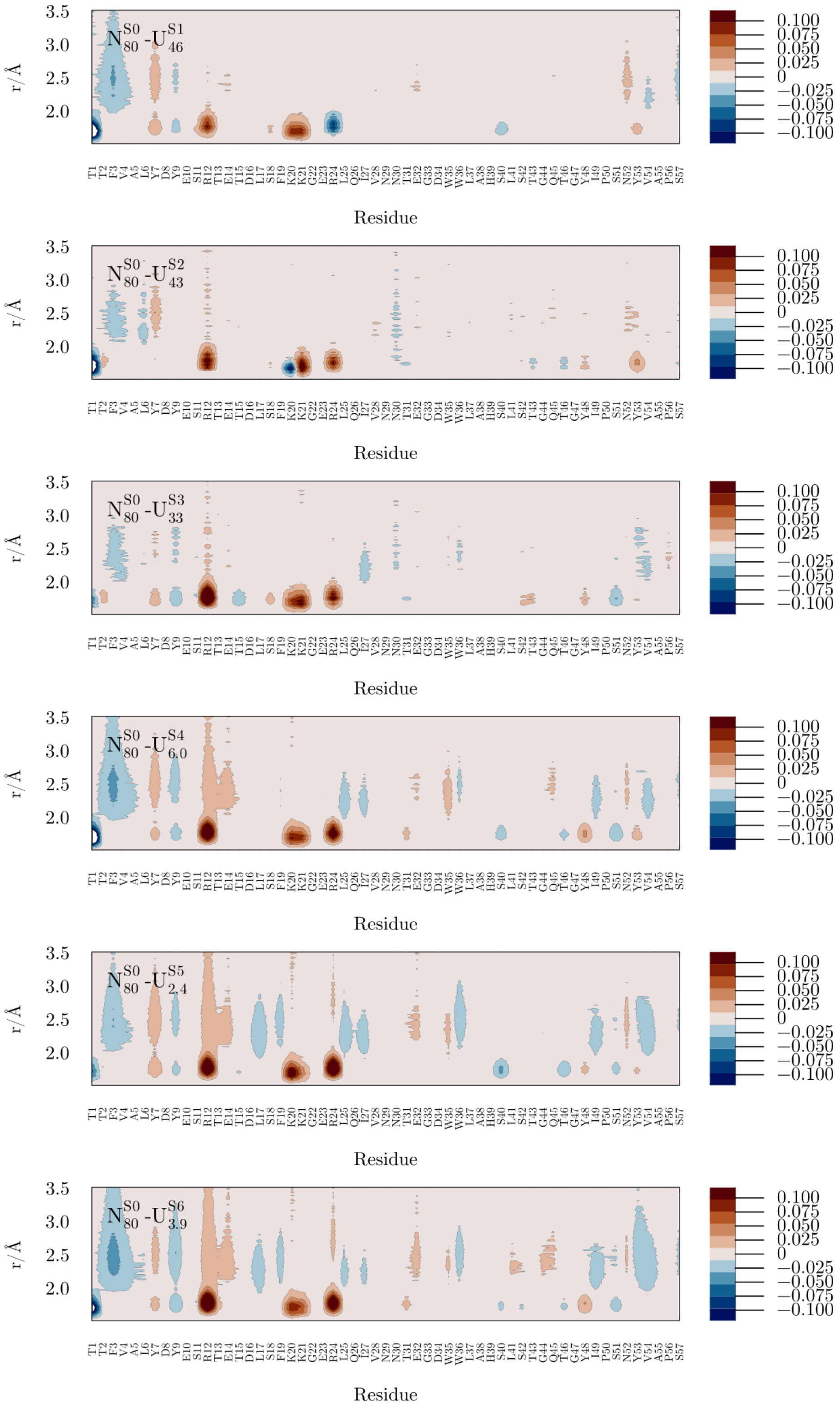


**Figure S10**. Differential density maps per residue for SH3 in TMAO solution 0.5 mol L-1.

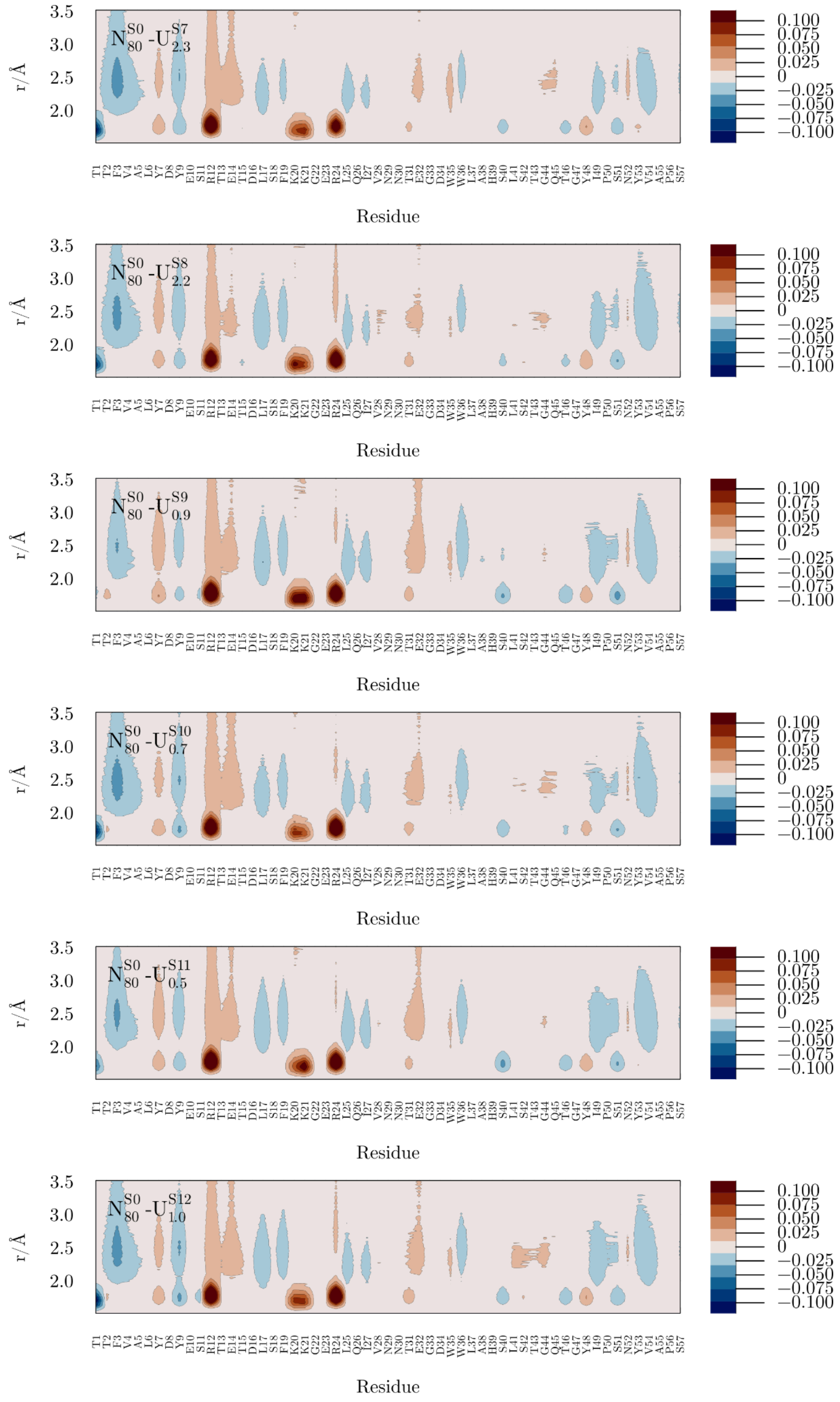


**Figure S10 (continued)**. Differential density maps per residue for SH3 in TMAO solution 0.5 mol $L^{-1}$.

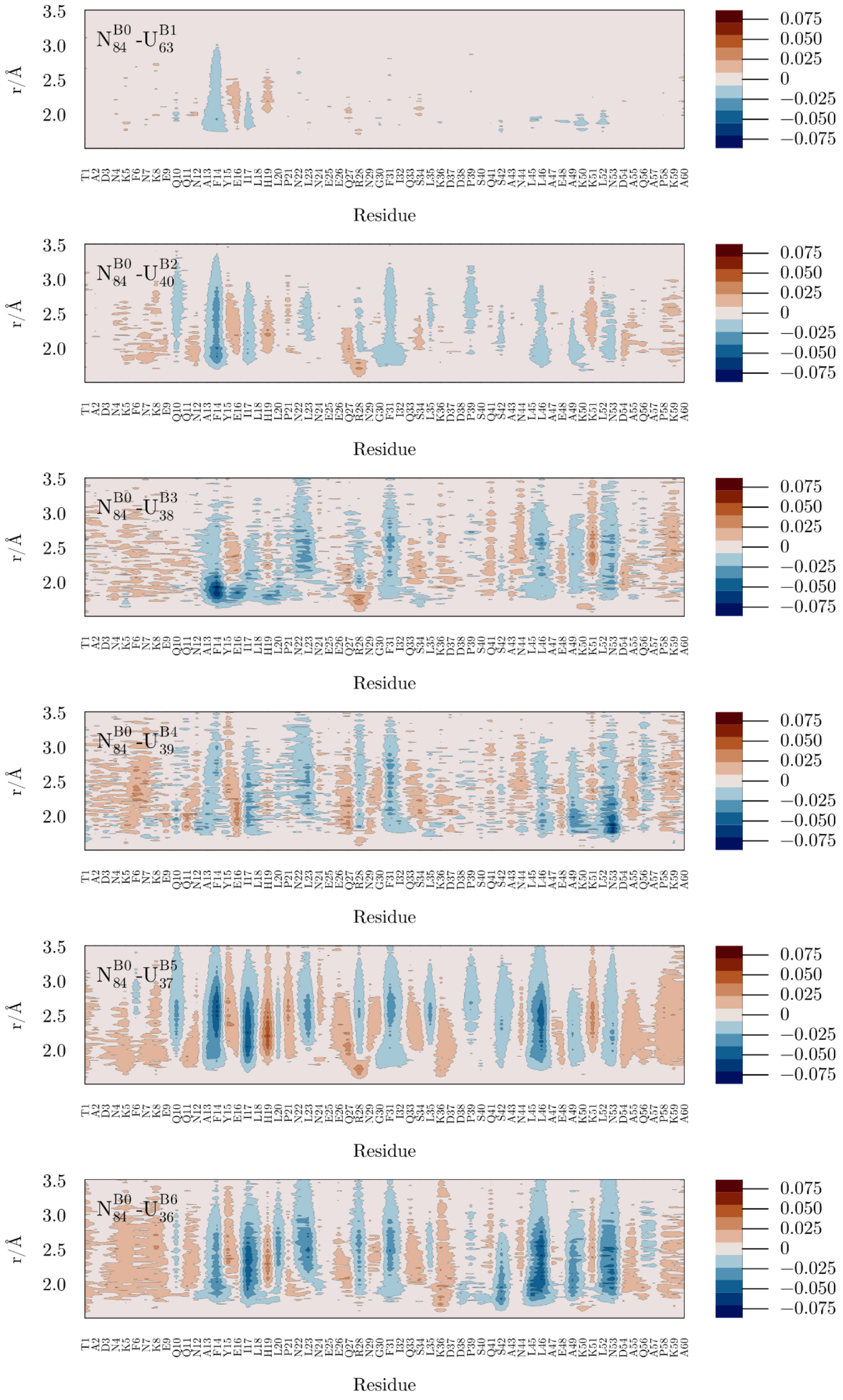


**Figure S11**. Differential density maps per residue for BdpA in urea solution 0.5 mol L-1.

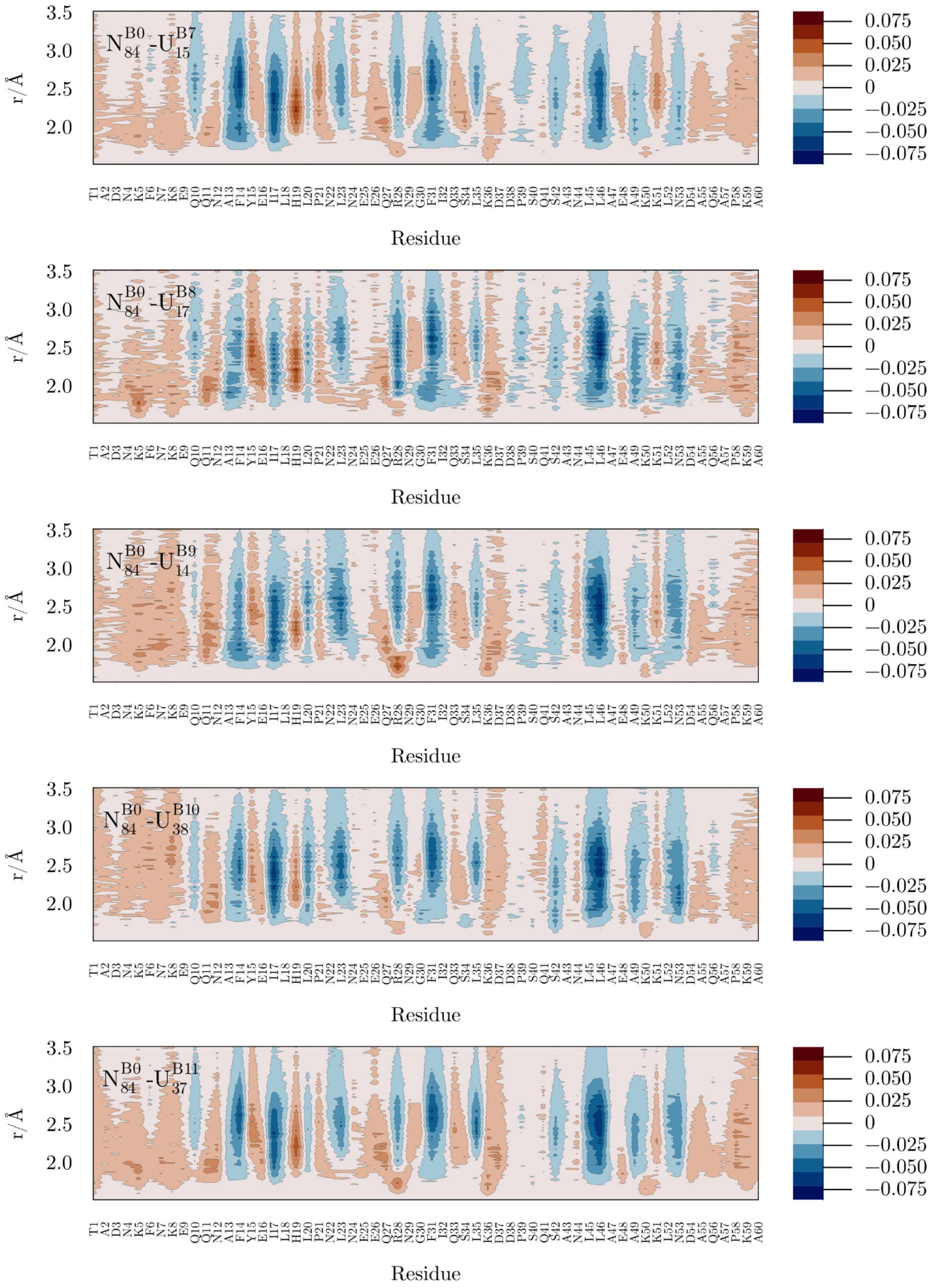


**Figure S11 (continued)**. Differential density maps per residue for BdpA in urea solution 0.5 mol $L^{-1}$.

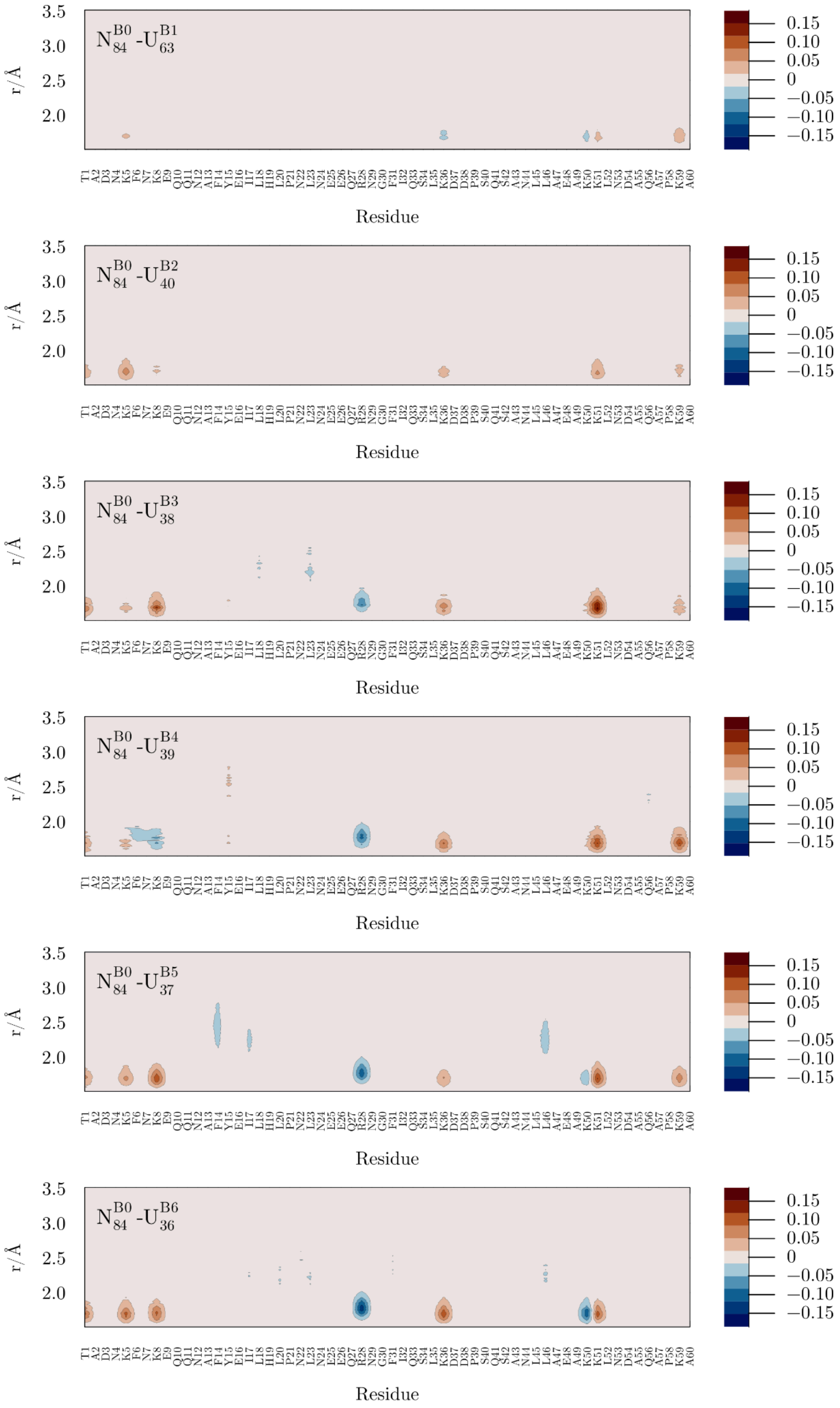


**Figure S12**. Differential density maps per residue for BdpA in TMAO solution 0.5 mol $L^{-1}$.

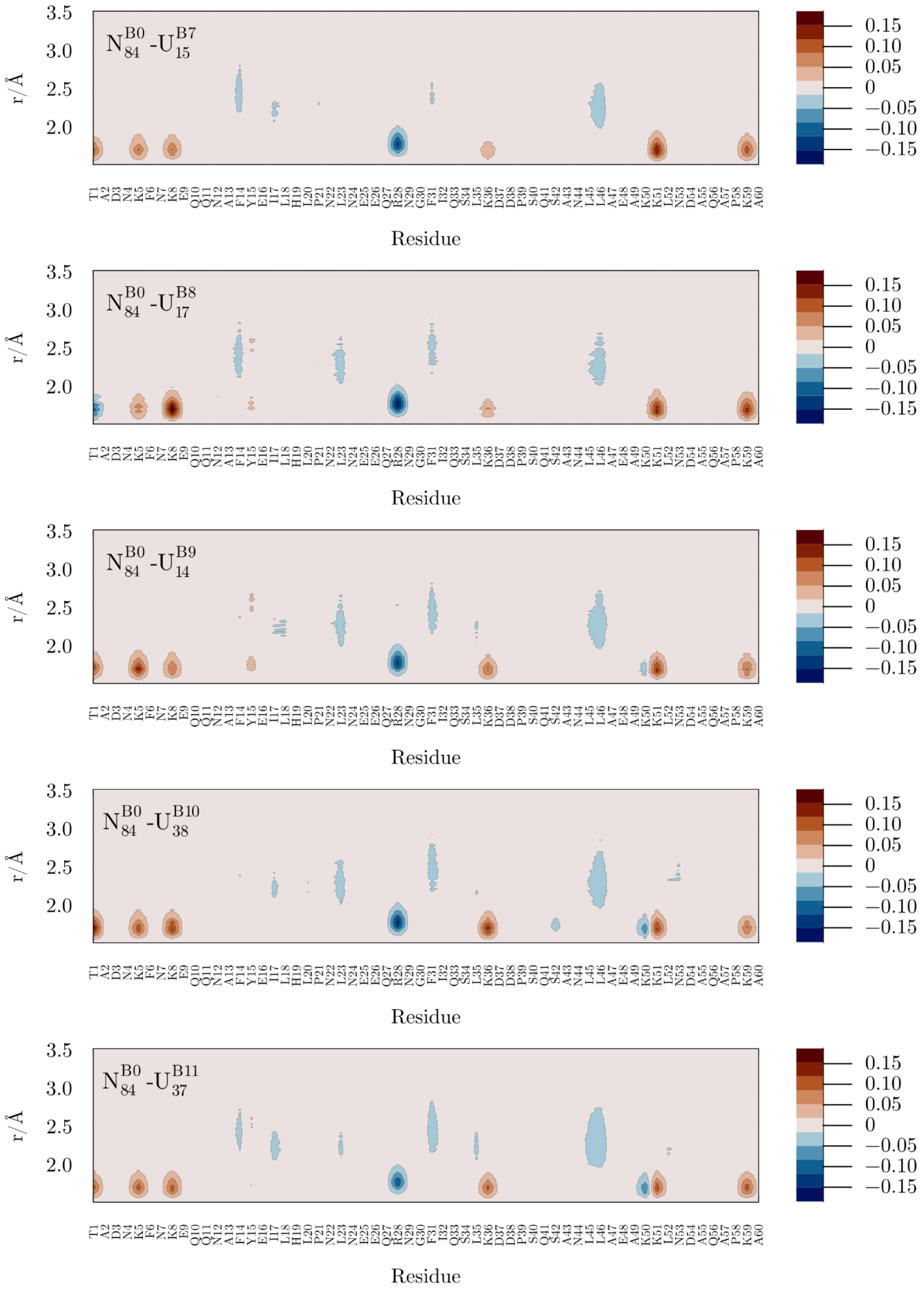


**Figure S12 (continued)**. Differential density maps per residue for BdpA in TMAO solution 0.5 mol $L^{-1}$.

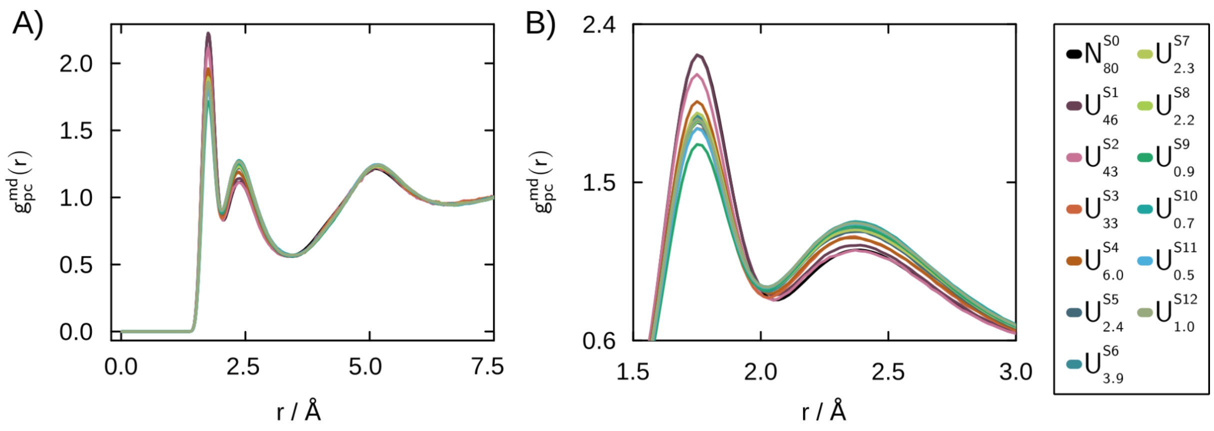


**Figure S13**. MDDF of TMAO 0.5 mol $L^{-1}$ for all ensembles of the SH3 protein.

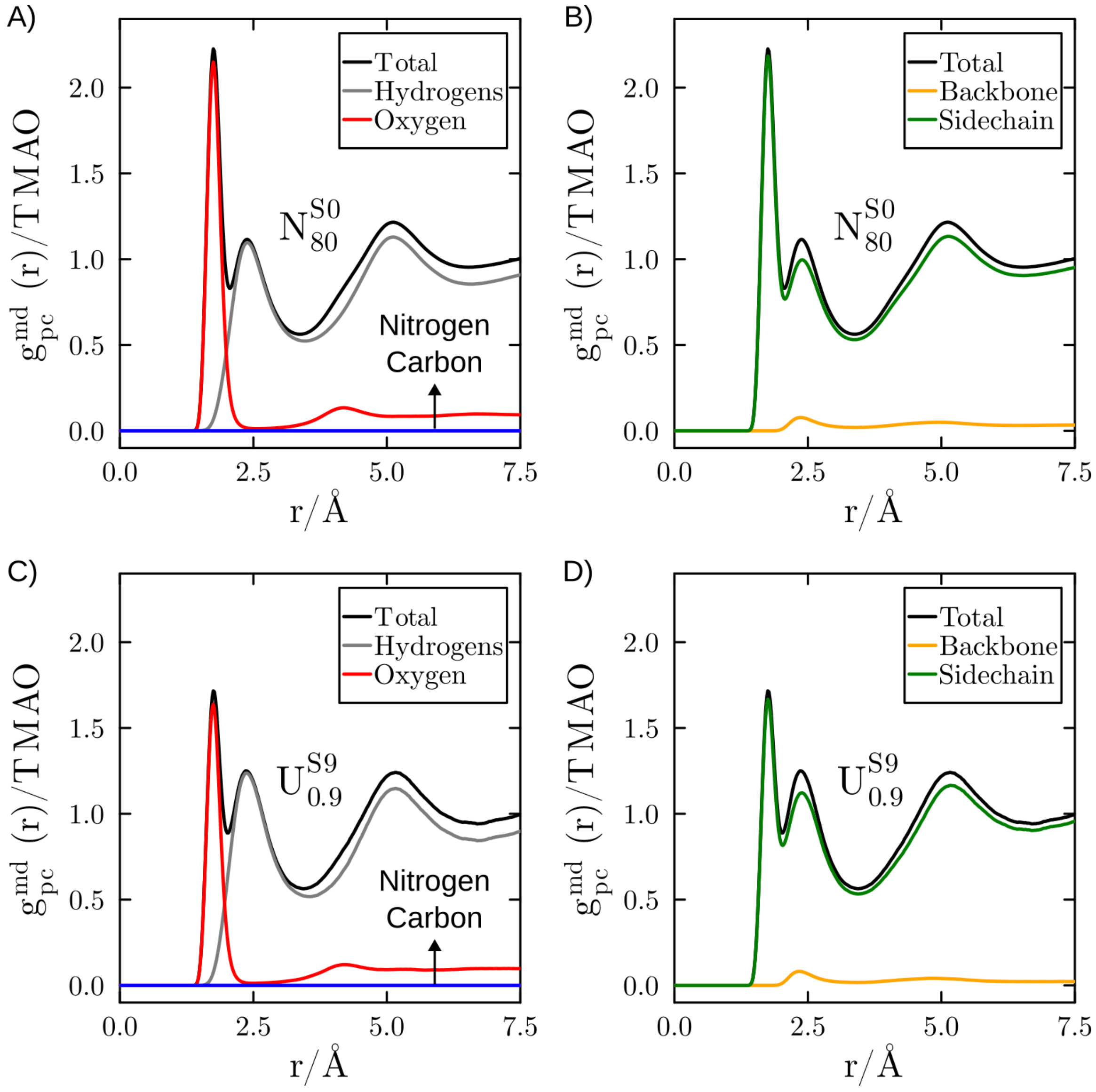


**Figure S14**. Total MDDF of TMAO and group contributions for the $N_{80}^{S0}$ and $U_{0.9}^{S9}$ ensembles. A) and C) show the contributions of the atoms and atom groups of the TMAO's MDDF of $N_{80}^{S0}$ and $U_{0.9}^{S9}$ ensembles. B) and D) show the respective contributions of the backbone (yellow) and side chain (green) to the total TMAO's MDDF.

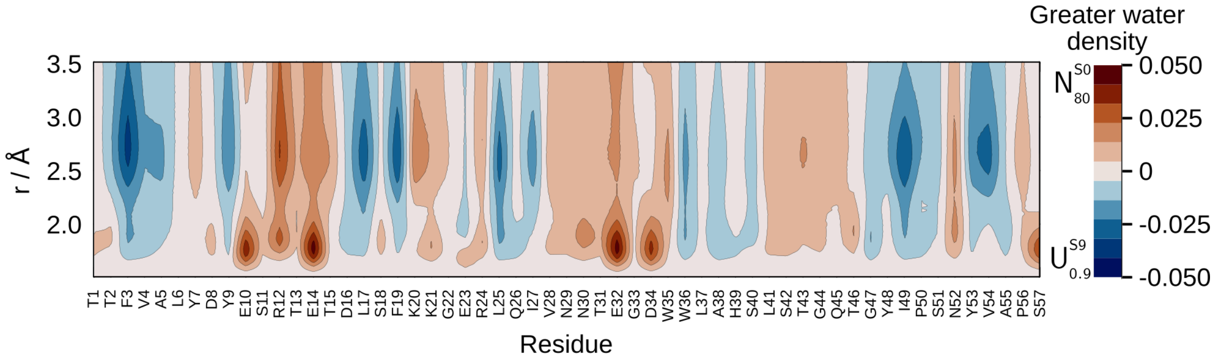


**Figure S15**. Difference in the MDDF density of the water in the vicinity of $N^{S0}_{80}$ and unfolded ($U^{S9}_{0.9}$) state in the TMAO 0.5 mol L$^{-1}$ solution. Red regions indicate higher water density near the $N^{S0}_{80}$ state, while blue regions show higher density around the $U^{S9}_{0.9}$ state. This pattern emphasizes the increased interactions of the solvent with mostly hydrophobic residues in the $U^{S9}_{0.9}$ state that are typically protected from the solvent in the $N^{S0}_{80}$ conformation.

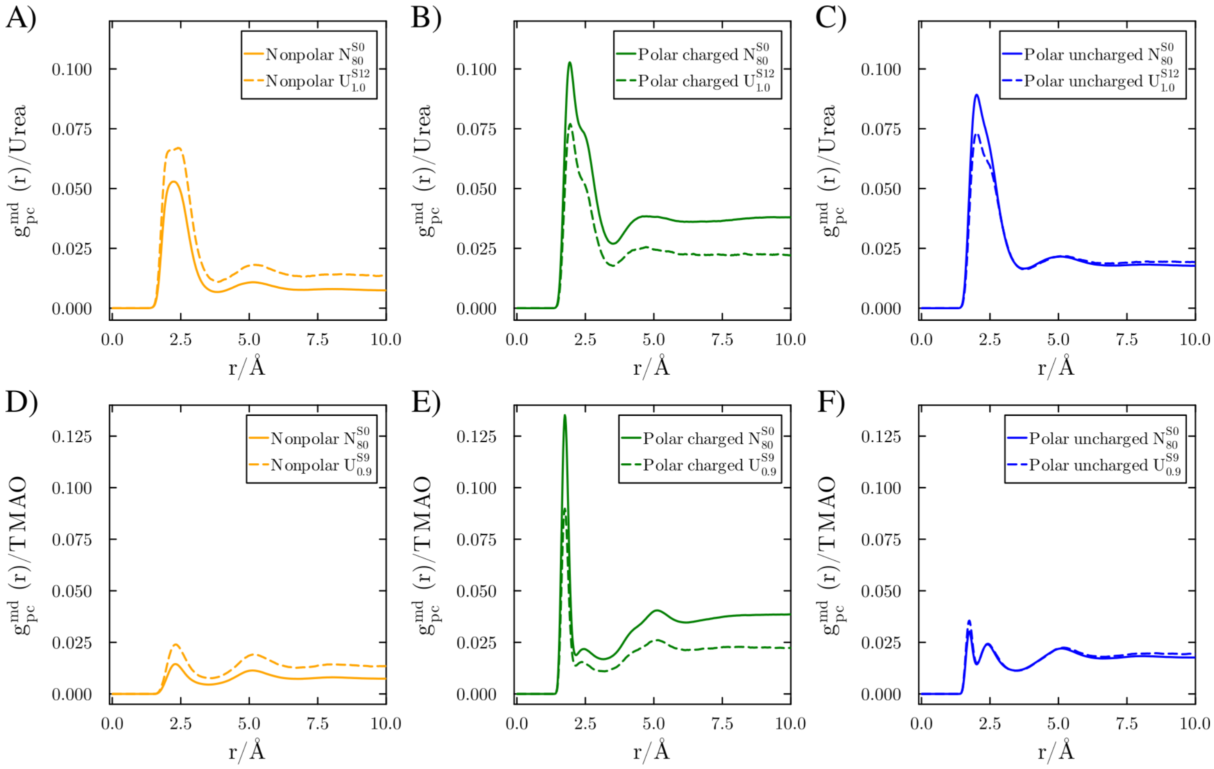


**Figure S16**. Normalized contributions of the MDDF for the SH3 protein, relative to the number of residues of each class. Panels A)-C) show the native (solid line) and unfolded (dotted line) ensembles in 0.5 M urea, and panels D)-F) show the corresponding ensembles in 0.5 M TMAO. Panels A) and D) correspond to nonpolar residues, B) and E) to polar charged residues, and C) and F) to polar uncharged residues.

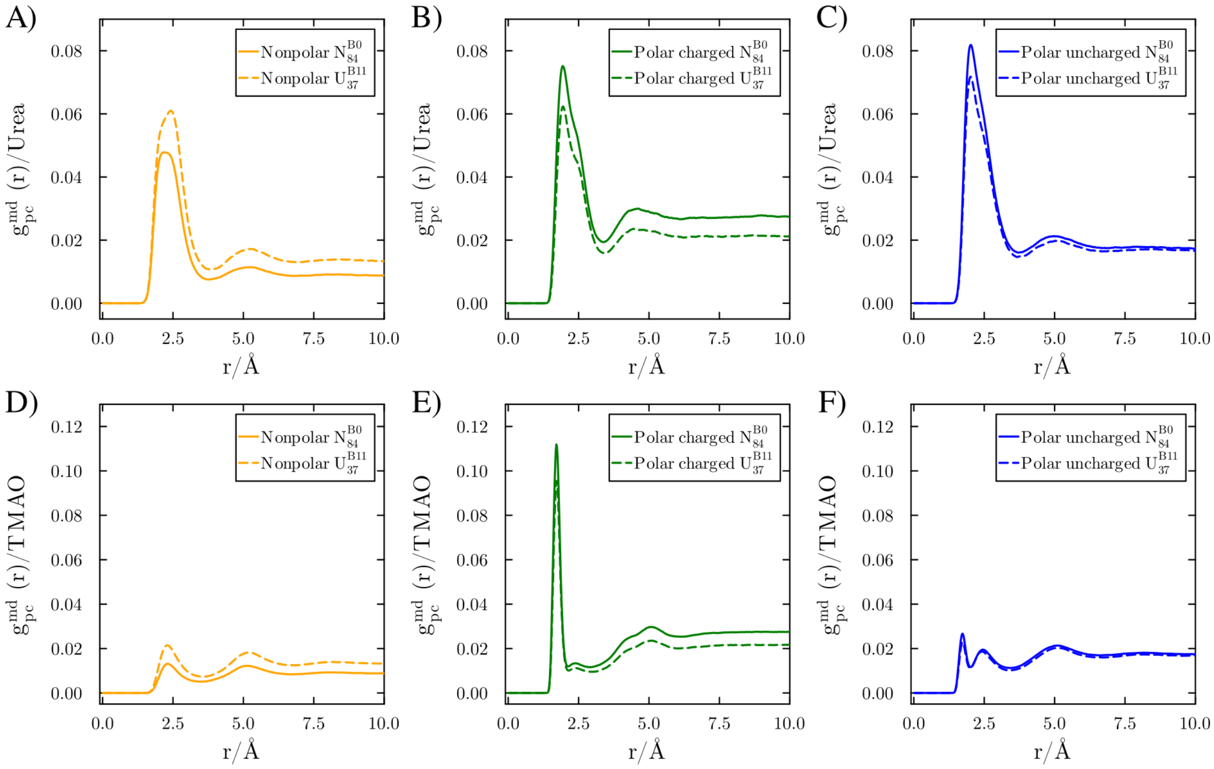


**Figure S17**. Contributions of the MDDF for the BdpA protein normalized by the abundance of residues of each class. Panels A)-C) show the native (solid line) and unfolded (dotted line) ensembles in 0.5 M urea, and panels D)-F) show the corresponding ensembles in 0.5 M TMAO. Panels A) and D) correspond to nonpolar residues, B) and E) to polar charged residues, and C) and F) to polar uncharged residues.

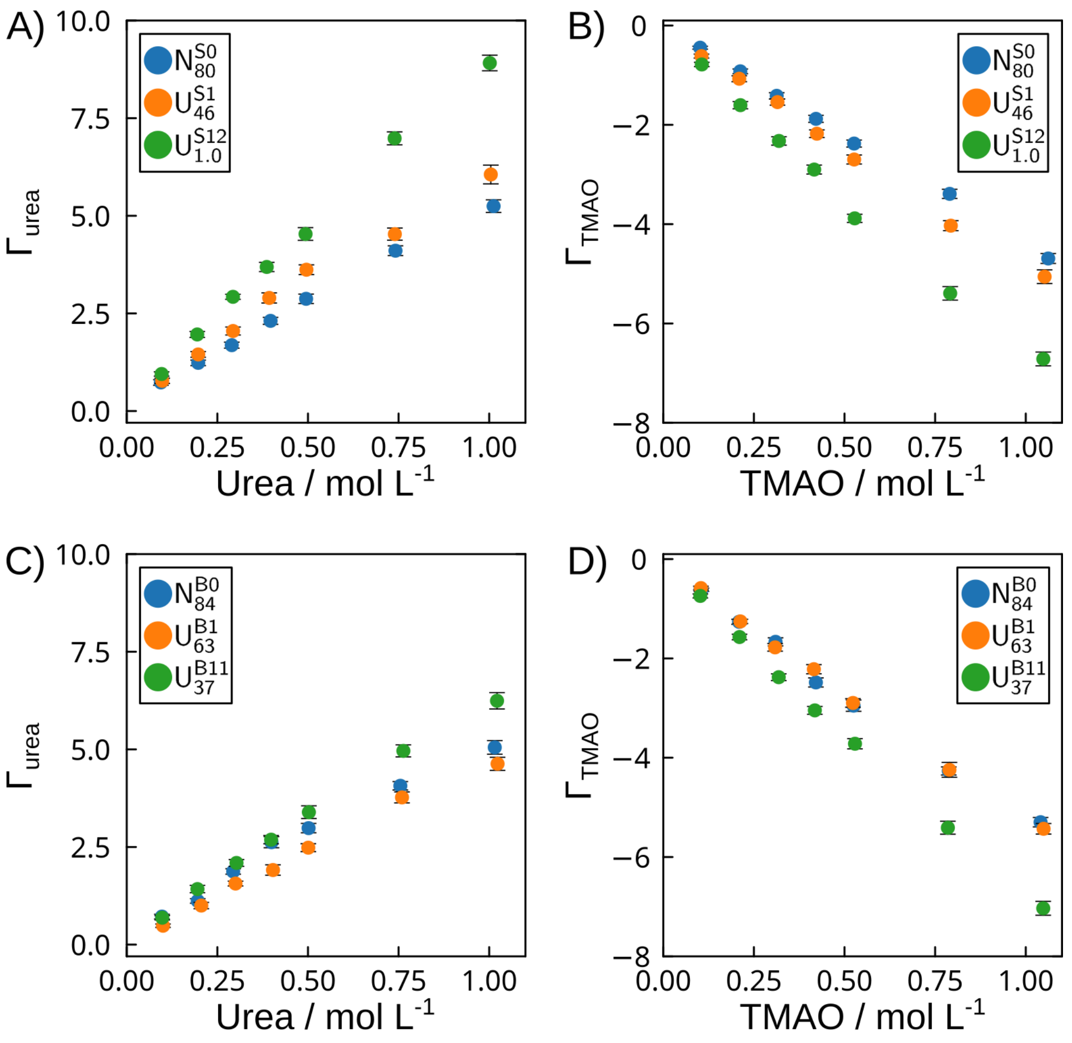


**Figure S18**. Preferential interaction parameters (Γs) as a function of the osmolyte concentrations. A) and B) show the Γ of Urea and TMAO for selected SH3 ensembles, respectively. Similarly, C) and D) show the Γ of Urea and TMAO for selected BdpA ensembles.

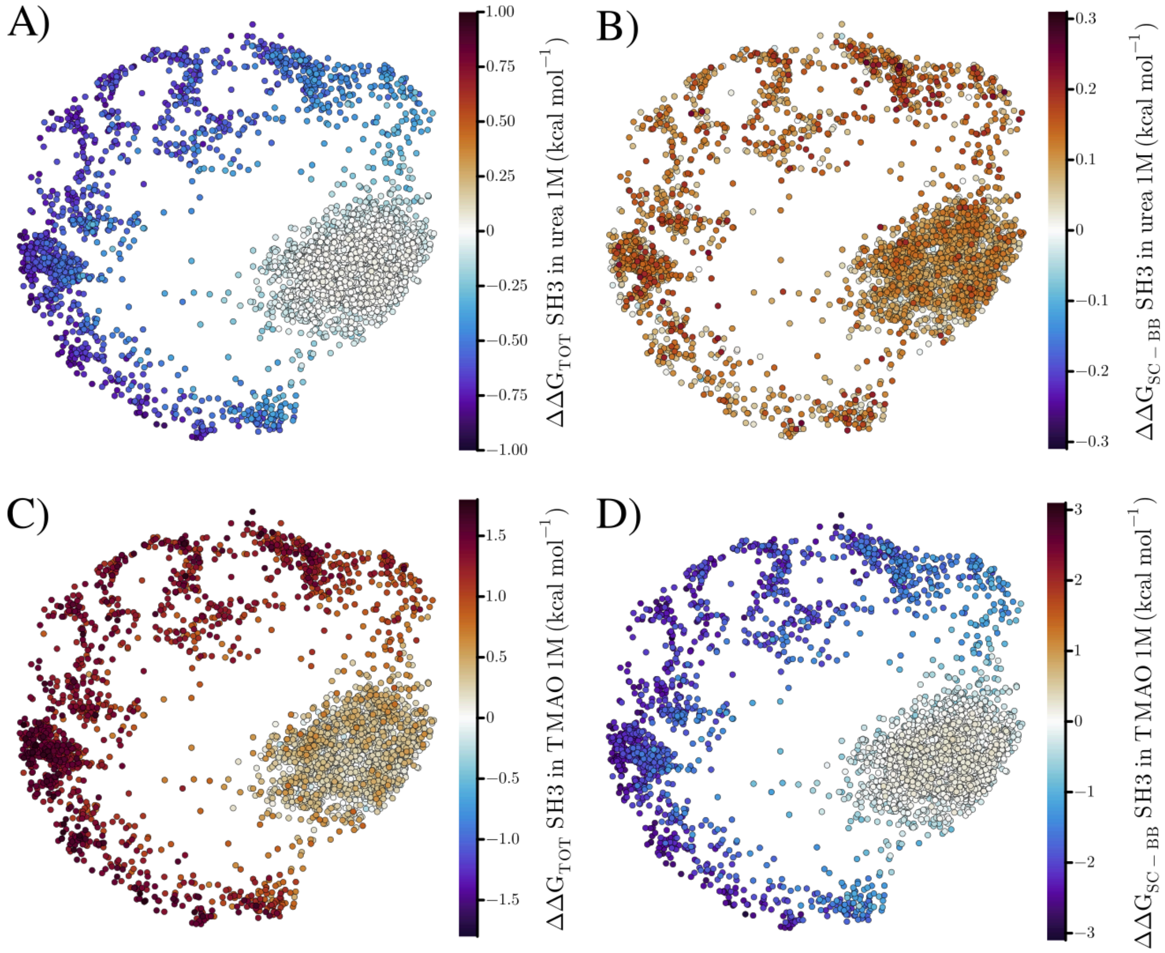


**Figure S19**. Folding ensemble of SH3 represented by the ELViM projection. Each dot corresponds to one structure, colored according to the transfer free energy (TFE) of SH3 in (A) urea and (C) TMAO (1 mol $L^{-1}$), relative to the native state, as predicted by Moeser and Horinek[2] and Auton and Bolen[3,4] models, respectively. Panels (B) and (D) display the corresponding differences in side chain and backbone contributions to the TFE.

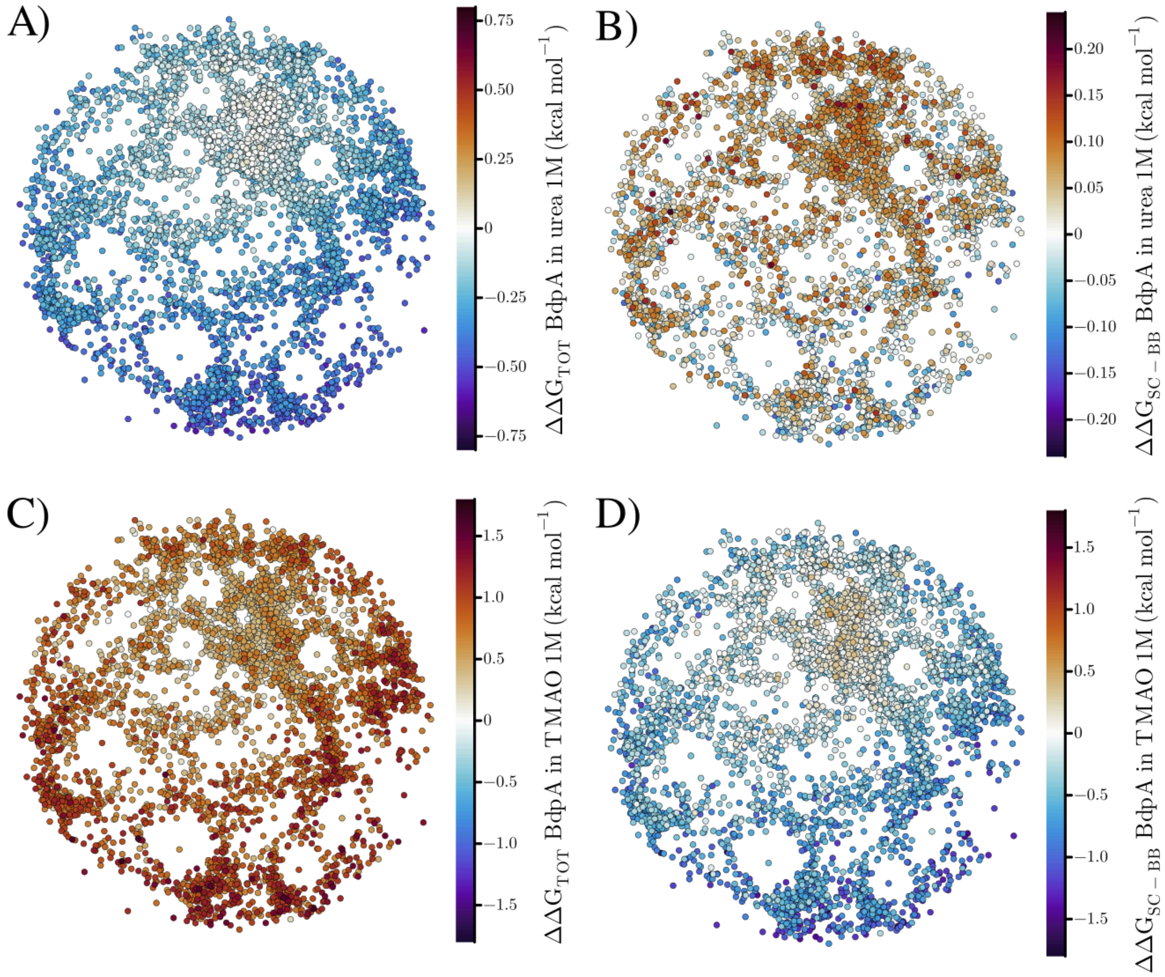


**Figure S20**. Folding ensemble of BdpA represented by the ELViM projection. Each dot corresponds to one structure, colored according to the transfer free energy (TFE) of SH3 in (A) urea and (C) TMAO (1 mol $L^{-1}$) relative to the native state, as predicted by Moeser and Horinek[2] and Auton and Bolen[3,4] models, respectively. Panels (B) and (D) display the corresponding differences in side chain and backbone contributions to the TFE.

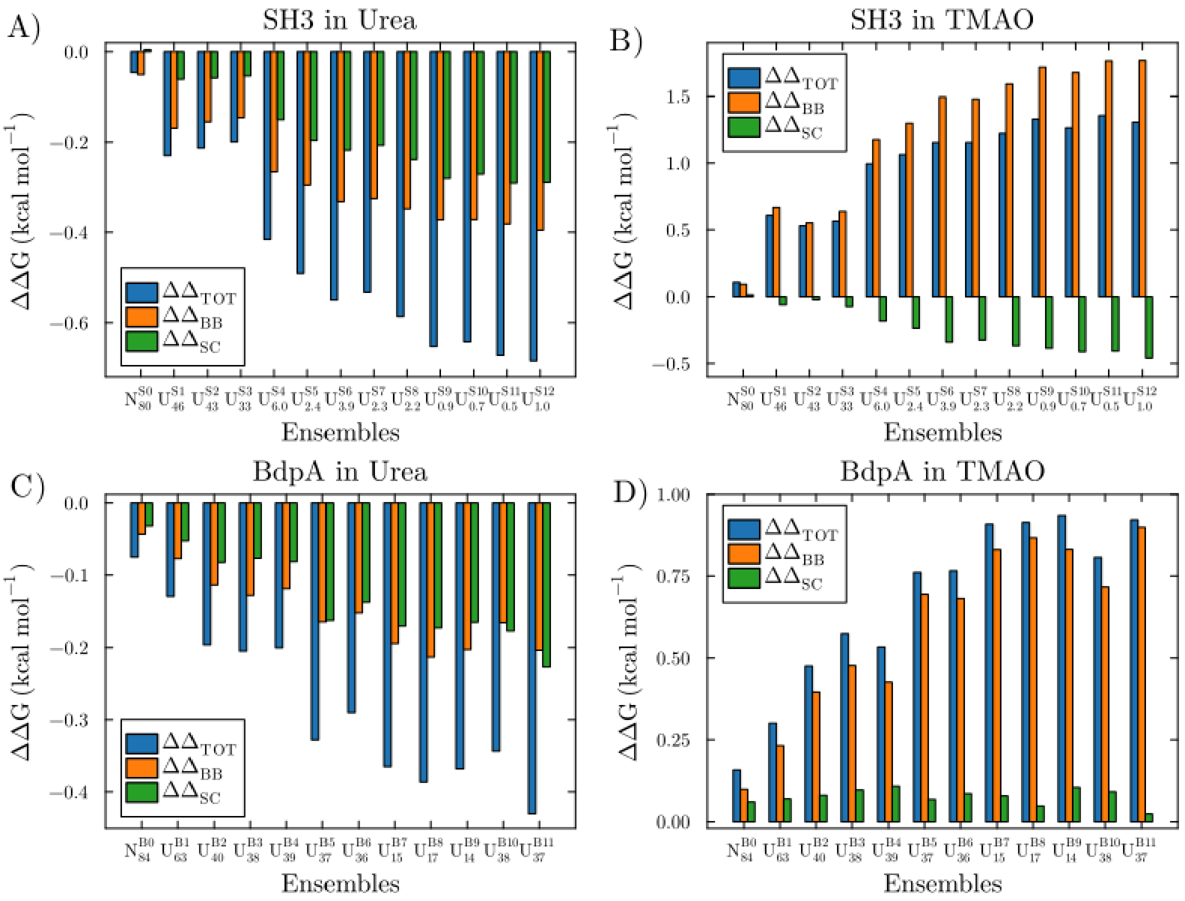


**Figure S21**. Decomposition of the predicted transfer free energy (ΔΔG) for SH3 and BdpA protein ensembles in 0.5 M urea[2] and 0.5 M TMAO.[3,4] The panels show the results for (A) SH3 in Urea, (B) SH3 in TMAO, (C) BdpA in Urea, and (D) BdpA in TMAO. Within each panel, bars represent the total $\Delta\Delta G_{TOT}$ (in blue) and its constituent contributions from the protein backbone ($\Delta\Delta G_{BB}$, in orange) and side chains ($\Delta\Delta G_{SC}$, in green). The x-axis denotes different conformational ensembles, from the native state (N) to unfolded states (U). All energy values are in kcal mol$^{-1}$.

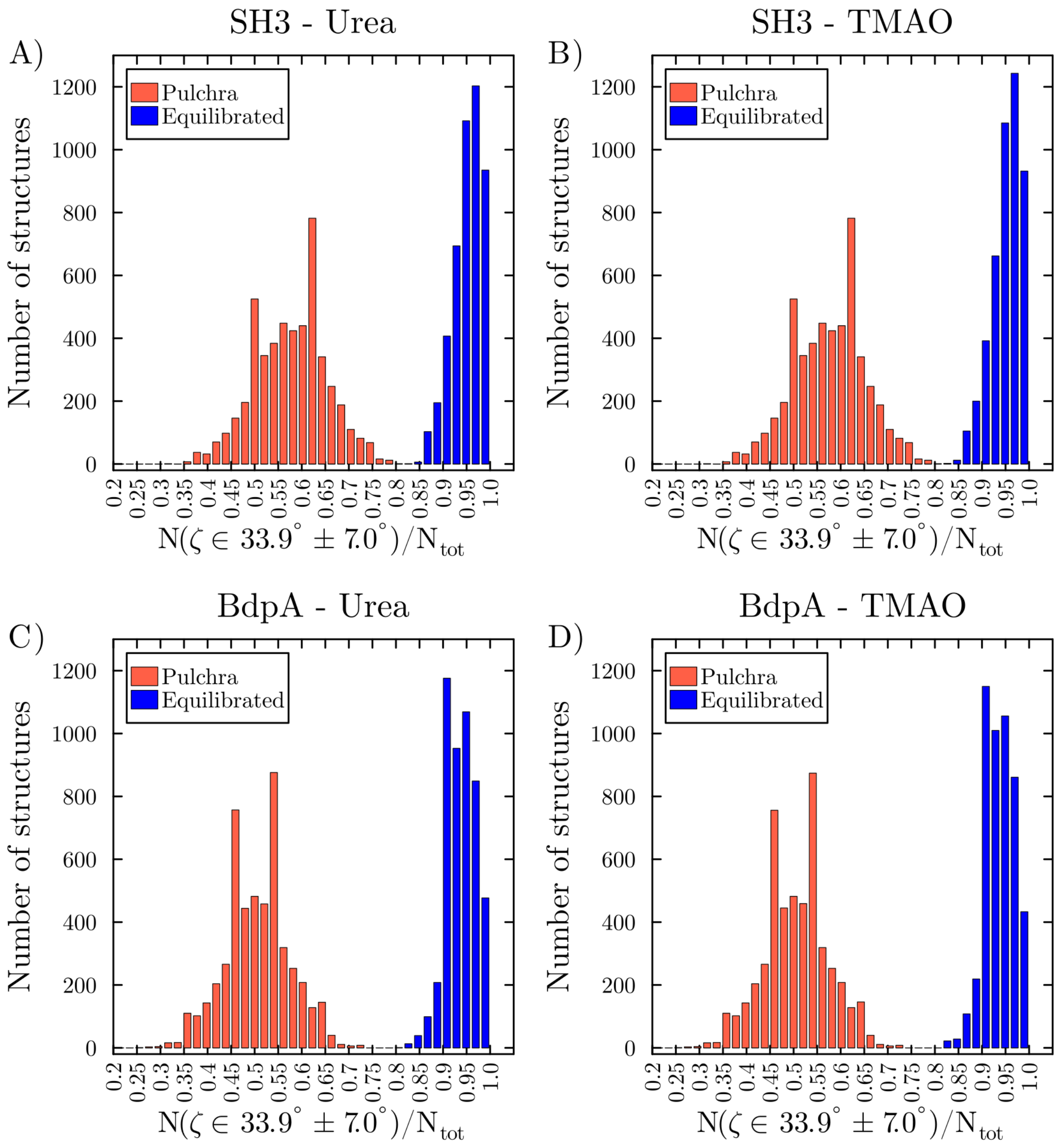


**Figure S22.** Distribution of the quality of the structures reconstructed from SBMs with Pulchra before (red) and after (blue) equilibration. For each structure, the fraction of residues displaying a virtual torsion angle $\zeta$ (C$\alpha$-N-C-C$\beta$) within 33.9° ± 7.0° was computed to evaluate the chirality of C$\alpha$ atoms. The values correspond to the mean and two standard deviations of the distributions observed in high-quality experimental protein models.[1] The equilibrated distributions, sharply distributed above 0.9, demonstrate that the all-atom reconstruction and equilibration were successful.

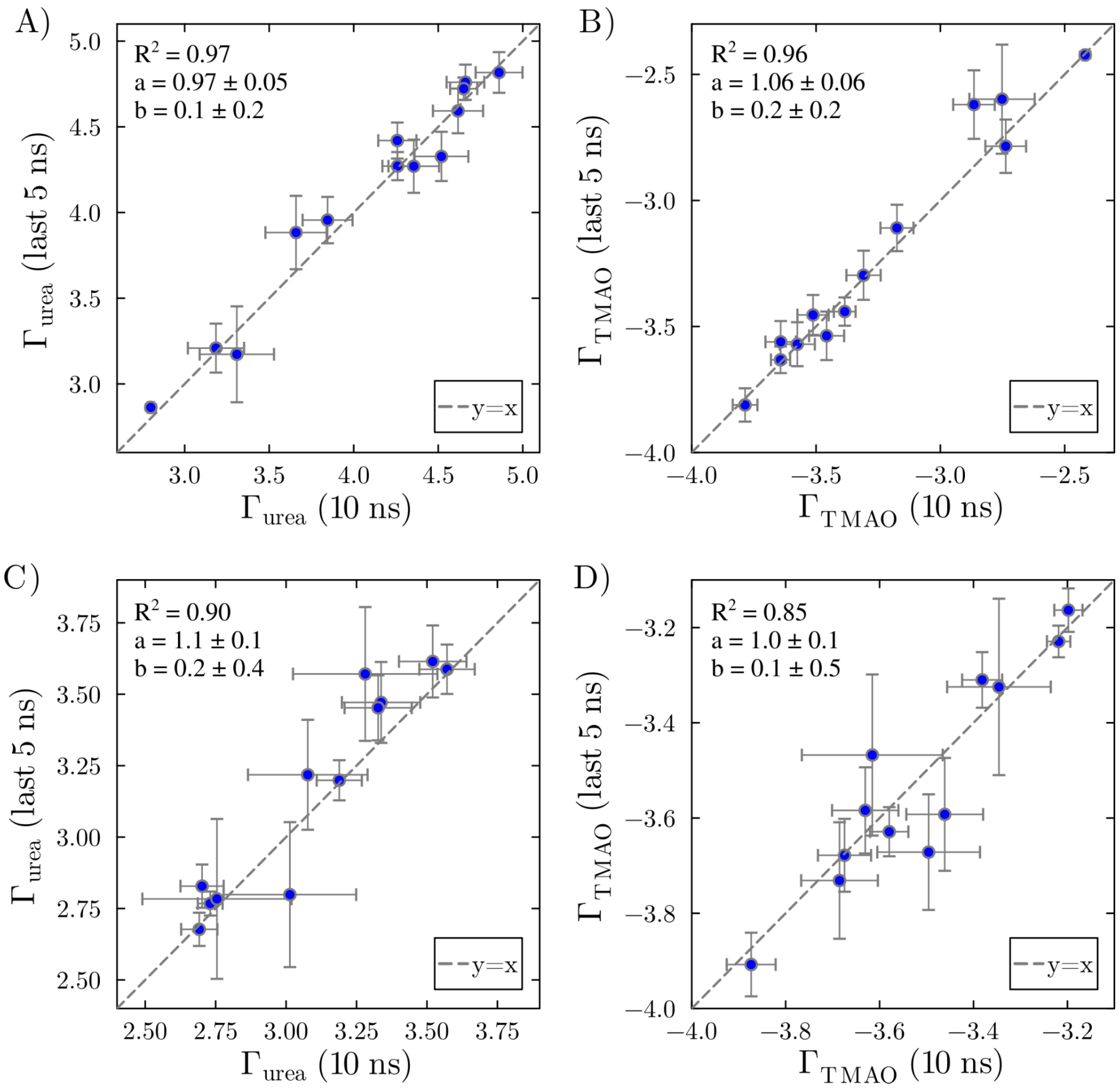


**Figure S23**. Preferential interaction parameters computed using only the last 5 ns of the simulations vs. those computed using the full 10 ns of simulations.

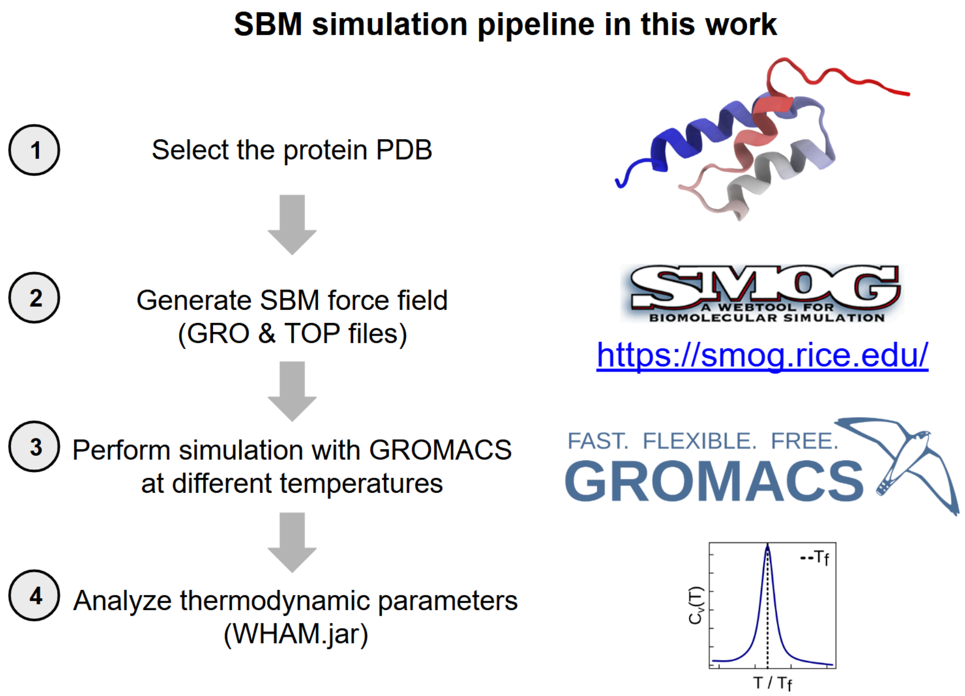


**Figure S24**. Short SMOG pipeline used in this study for the SBM simulations.

## References


(1) Morris, A. L.; MacArthur, M. W.; Hutchinson, E. G.; Thornton, J. M. Stereochemical Quality of Protein Structure Coordinates. *Proteins* **1992**, *12* (4), 345–364.

(2) Moeser, B.; Horinek, D. Unified Description of Urea Denaturation: Backbone and Side Chains Contribute Equally in the Transfer Model. *J Phys Chem B* **2014**, *118* (1), 107–114.

(3) Auton, M.; Bolen, D. W. Application of the Transfer Model to Understand How Naturally Occurring Osmolytes Affect Protein Stability. *Methods Enzymol* **2007**, *428*, 397–418.

(4) Auton, M.; Holthauzen, L. M. F.; Bolen, D. W. Anatomy of Energetic Changes Accompanying Urea-Induced Protein Denaturation. *Proc Natl Acad Sci U S A* **2007**, *104* (39), 15317–15322.
